# On the Precise Tuning of Optical Filtering Features in Nanoporous Anodic Alumina Distributed Bragg Reflectors

**DOI:** 10.1038/s41598-018-22895-5

**Published:** 2018-03-15

**Authors:** Cheryl Suwen Law, Siew Yee Lim, Abel Santos

**Affiliations:** 1 School of Chemical Engineering, The University of Adelaide, Adelaide, SA 5005 Australia; 2 Institute for Photonics and Advanced Sensing (IPAS), The University of Adelaide, 5005 Adelaide, Australia; 3 ARC Centre of Excellence for Nanoscale BioPhotonics (CNBP), The University of Adelaide, 5005 Adelaide, Australia

## Abstract

This study presents a nanofabrication approach that enables the production of nanoporous anodic alumina distributed Bragg reflectors (NAA-DBRs) with finely engineered light filtering features across the spectral regions. The photonic stopband (PSB) of these NAA-based photonic crystal (PC) structures is precisely tuned by an apodization strategy applied during stepwise pulse anodization with the aim of engineering the effective medium of NAA-DBRs in depth. We systematically assess the effect of different fabrication parameters such as apodization function (i.e. linear positive, linear negative, logarithmic positive and logarithmic negative), amplitude difference (from 0.105 to 0.420 mA cm^−2^), current density offset (from 0.140 to 0.560 mA cm^−2^), anodization period (from 1100 to 1700 s), and pore widening time (from 0 to 6 min) on the quality and central wavelength of the PSB of NAA-DBRs. The PSB’s features these PC structures are demonstrated to be highly tunable with the fabrication parameters, where a logarithmic negative apodization is found to be the most effective function to produce NAA-DBRs with high quality PSBs across the UV-visible-NIR spectrum. Our study establishes that apodized NAA-DBRs are more sensitive to changes in their effective medium than non-apodized NAA-DBRs, making them more suitable sensing platforms to develop advanced optical sensing systems.

## Introduction

Photonic crystals (PCs) in various forms such as optical lenses, fibers, mirrors, resonators, cavities, thin films, and optical filters are key elements in advanced optical devices since these structures enable the precise control over electromagnetic waves by light–matter interactions at the nanoscale. Of all the PC structures, optical filters are vital components for a broad range of applications, including laser mirrors, arc welding, polarizers, solar protection, photography, imaging, astronomy, and complex instrumentation^1,2^. PCs can selectively allow and forbid the pass of light of certain wavelengths or energies when photons travel across the PCs’ structure. This property can be precisely designed by engineering the PCs’ structure to develop PC-based advanced optical filters with unique light-filtering features^3^. Typically, optical filters are fabricated by chemical vapor deposition^3,4^, photolithography^5^, and co-evaporation^6^, processes through which substrates such as thin glass, plastic, and dielectric materials (e.g. silicon dioxide, titanium dioxide, zinc sulfide, magnesium fluoride) are endowed with selective light-filtering properties^3^. The raise of nanotechnology has enabled multiple opportunities to produce advanced PC-based optical filters using nanomaterials featuring layers of alternating refractive index. Among these, PC structures based on nanoporous materials produced by electrochemical etching of metals have opened new opportunities to develop advanced photonic platforms with applicability in photonics, photocatalysis, optoelectronics, and sensing^7^. The refractive index/dielectric constant of these nanomaterials can be engineered a 1D, 2D, or 3D fashion by modulating their porosity in depth, enabling the generation of a broad range of multi-dimensional PCs such as Bragg reflectors^8,9^, microcavities^10,11^, waveguides^12,13^, and others^14,15^.

Of all these PCs, nanoporous anodic alumina photonic crystals (NAA-PCs) produced by electrochemical oxidation (anodization) of aluminum have attracted considerable attention during recent years due to the versatility of the nanoporous structure of NAA^16^. This top-down nanofabrication approach offers industrial scalability (from mm^2^ to m^2^), cost-competitiveness, and versatile control over the features of nanopores, which can be modulated with precision by means of the anodization parameters to generate unique multi-dimensional PC structures able to guide, reflect, modulate, confine, transmit, emit, and enhance incident light selectively across the spectral regions^17,18^. Recent studies have demonstrated that rationally designed pulse-like anodization profiles under suitable conditions enable the precise engineering of the photonic stopband (PSB) of NAA-PCs by creating structures such as gradient index filters^19–22^, optical microcavities^23^, distributed Bragg reflectors^24–28^, bandpass and linear variable bandpass filters^29,30^. These PCs can be used as optical filters for a plethora of applications due to the flexibility and selectively to engineer their light-filtering features across the spectral regions^31,32^.

**Figure 1:**
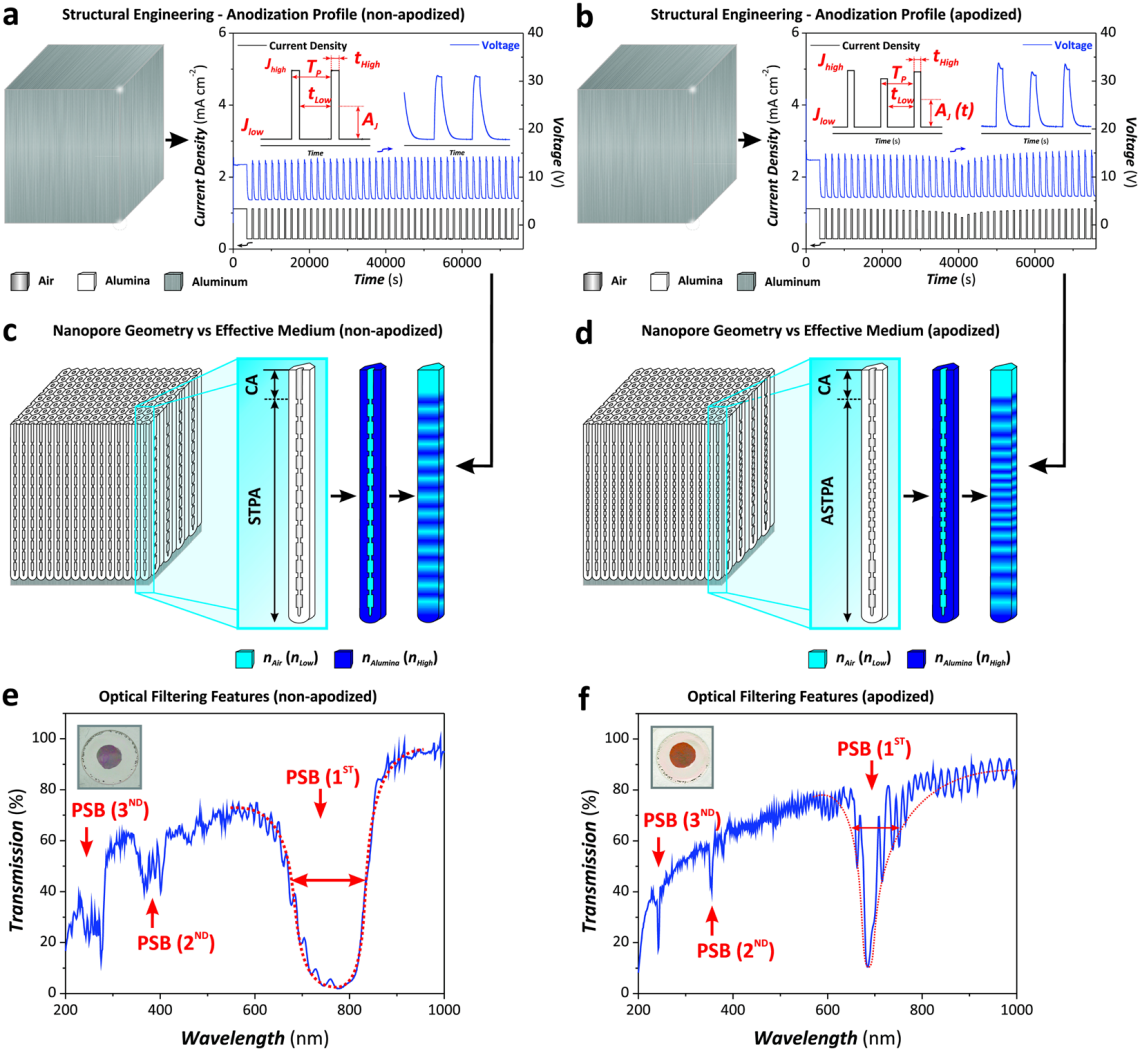
Conceptual illustration showing the generation of nanoporous anodic alumina distributed Bragg reflectors (NAA-DBRs) by stepwise pulse anodization (STPA) and the apodization of optical signals by apodized stepwise pulse anodization (ASTPA). (**a** and **b**) Representative STPA and ASTPA profiles used to fabricate NAA-DBRs (left – aluminum substrates; right – real anodization profiles with insets showing graphical descriptions of the anodization parameters: *T*_*P*_ = anodization period, *A*_*J*_ = anodization amplitude (constant for STPA and time-dependent for ASTPA), *J*_*High*_ and *J*_*Low*_ = high and low current density values, respectively, *t*_*High*_ and *t*_*Low*_ = anodization time at *J*_*High*_ and *J*_*Low*_, respectively) (Note: CA = constant current anodization step). (**c** and **d**) Scheme describing the correlation between nanopore geometry and distribution of high and low refractive indexes (*n*_*Alumina*_ = *n*_*High*_ ~1.7 and *n*_*Air*_ = *n*_*Low*_ ~1.0) in depth for non-apodized and apodized NAA-DBRs, respectively. (**e** and **f**) Representative transmission spectra of non-apodized (*T*_*P*_ = 1700 s, *A*_*J*_ = 0.420 mA cm^−2^, *J*_*min*_ = *J*_*offset*_ = 0.280 mA cm^−2^, *t*_*STPA*_ = 20 h, and *t*_*pw*_ = 0 min) and apodized (apodization function = logarithmic negative, *T*_*P*_ = 1700 s, Δ*A*_*J*_ = 0.210 mA cm^−2^, *J*_*min*_ = *J*_*offset*_ = 0.280 mA cm^−2^, *t*_*ASTPA*_ = 20 h, and *t*_*pw*_ = 0 min) NAA-DBRs with insets showing digital images of these photonic crystal structures, respectively.

In this study, we demonstrate for the first time that an apodized stepwise pulse anodization (STPA) approach enables the fine tuning of the features of the characteristic PSB of NAA-based distributed Bragg reflectors (DBRs) (Fig. 1). The effect of the fabrication parameters (i.e. apodization function, anodizing current density, amplitude difference, current density offset, anodization period, and pore widening time) on the characteristic PSB of NAA-DBRs is systematically analyzed to attain full controllability over the filtering features of these PCs and to establish the most optimal path for high quality PSBs. We further assess the effective medium of apodized and non-apodized NAA-DBRs after infiltrating their nanopores with mediums of different refractive index. Shifts in the position of the characteristic PSB of these PCs demonstrate that to apodize the structure of NAA-DBRs can enhance the sensitivity of these PCs, opening new opportunities to develop materials for sensing applications.

## Results and Discussion

### Apodization of Stepwise Pulse Anodization Profiles

**Figure 2:**
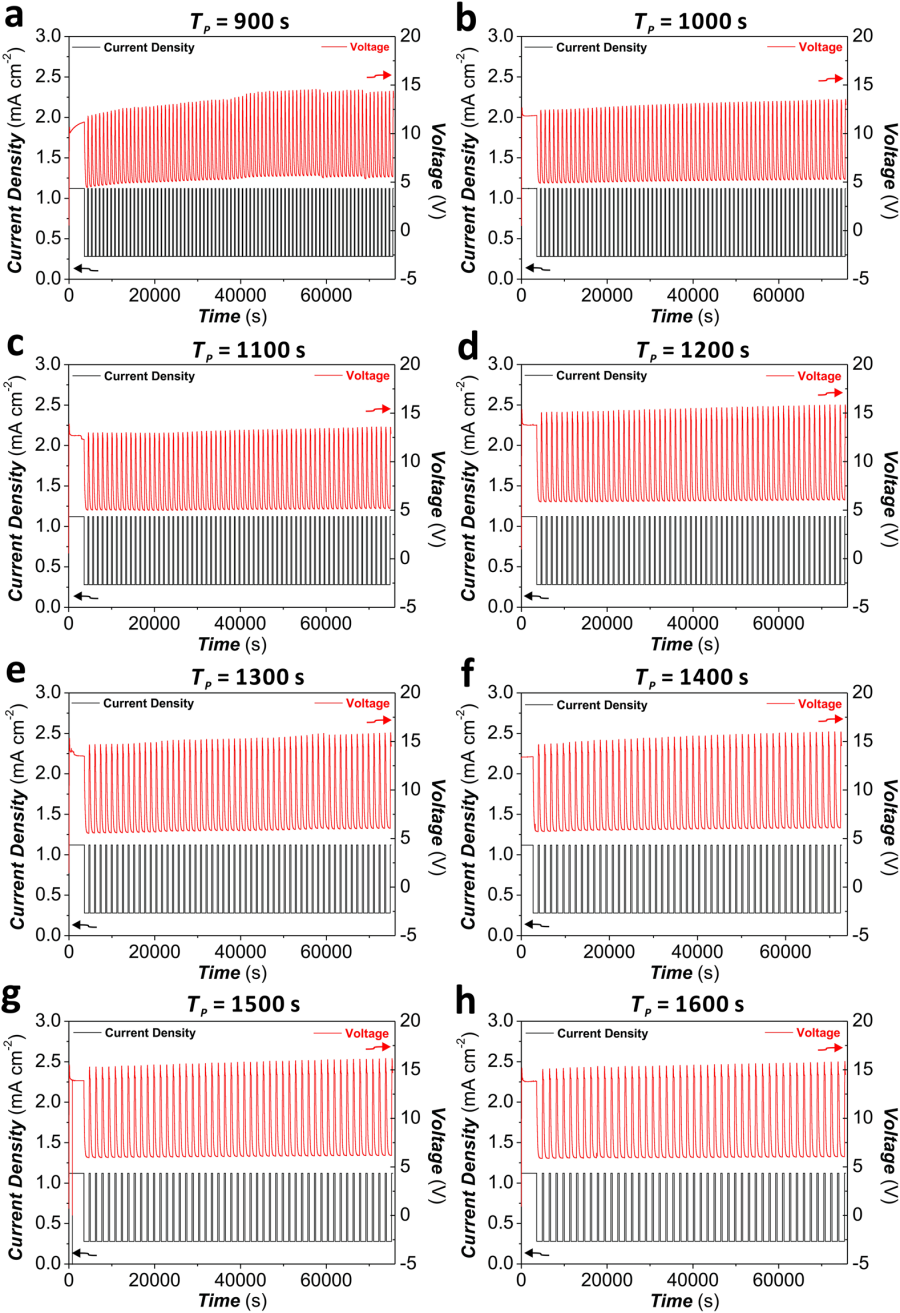
Representative STPA anodization profiles used to produce non-apodized nanoporous anodic alumina distributed Bragg reflectors (NAA-DBRs) by modifying the anodization period from *T*_*P*_ = 900 to 1700 s with *ΔT*_*P*_ = 100 s (constant parameters – *A*_*J*_ = 0.420 mA cm^−2^, *J*_*Low*_ = *J*_*Offset*_ = 0.280 mA cm^−2^, and *t*_*STPA*_ = 20 h). (**a**) *T*_*P*_ = 900 s. (**b**) *T*_*P*_ = 1000 s. (**c**) *T*_*P*_ = 1100 s. (**d**) *T*_*P*_ = 1200 s. (**e**) *T*_*P*_ = 1300 s. (**f**) *T*_*P*_ = 1400 s. (**g**) *T*_*P*_ = 1500 s. (**h**) *T*_*P*_ = 1600 s.

**Figure 3:**
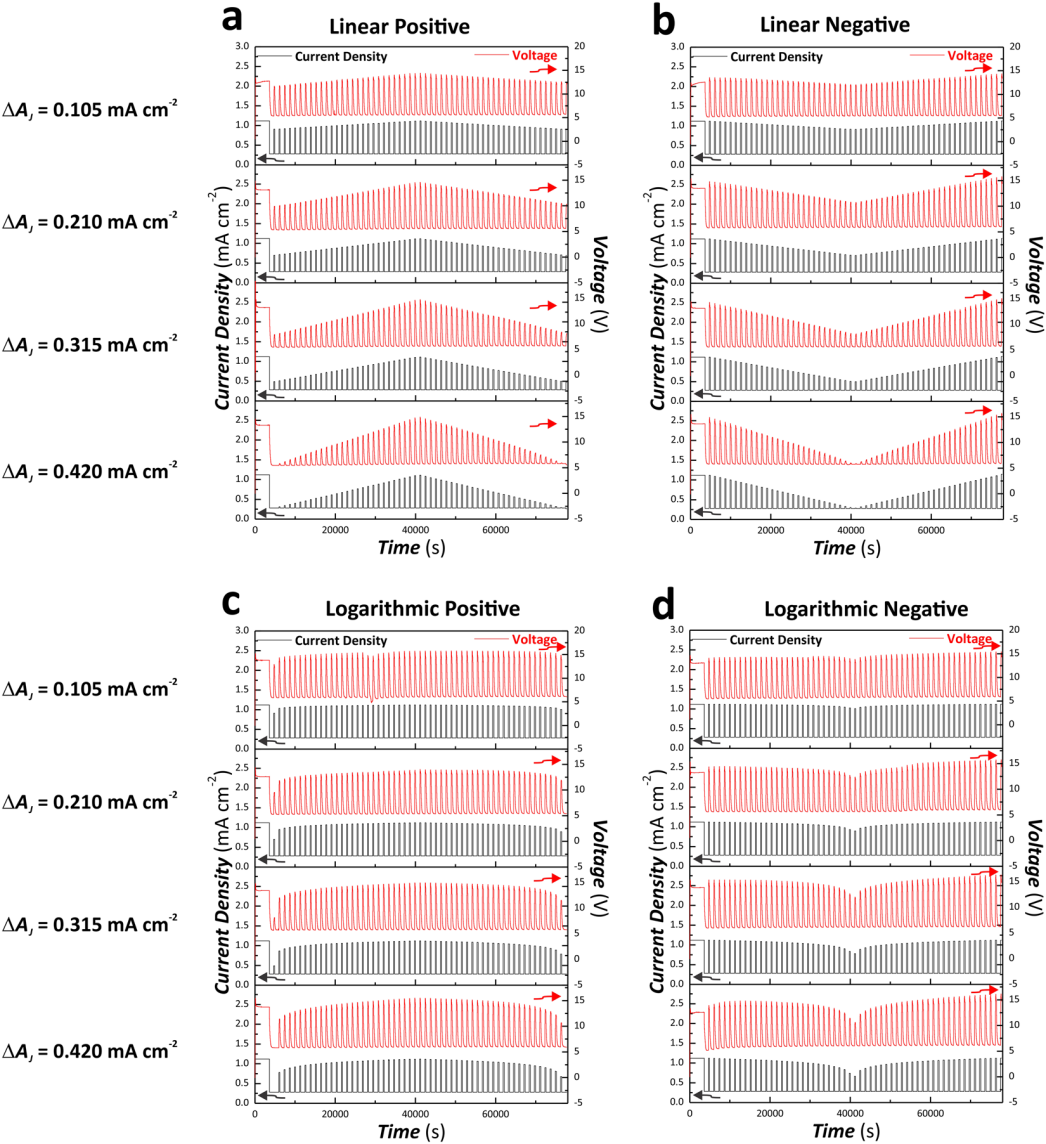
Representative ASTPA anodization profiles used to produce apodized nanoporous anodic alumina distributed Bragg reflectors (NAA-DBRs) by modifying the apodization function (linear positive, linear negative, logarithmic positive, and logarithmic negative) and the anodization amplitude difference from *ΔA*_*J*_ = 0.105 to 0.420 mA cm^−2^ (constant parameters – *T*_*P*_ = 1300 s, *J*_*Low*_ = *J*_*Offset*_ = 0.280 mA cm^−2^, and *t*_*ASTPA*_ = 20 h. (**a**) Linear positive apodization. (**b**) Linear negative apodization. (**c**) Logarithmic positive apodization. (**d**) Logarithmic negative apodization.

Figure 2 displays the anodization profiles of a set of non-apodized NAA-DBRs produced by STPA at different anodization periods (*T*_*p*_), from *T*_*P*_ = 900 s to 1700 s with *ΔT*_*P*_ = 100 s (Note: displayed only from 900 to 1600 s). These NAA photonic structures were produced by STPA approach under galvanostatic conditions, where the current density (*J*) is switched between maximum (*J*_*Max*_ = 1.120 mA cm^−2^) and minimum (*J*_*Min*_ = 0.280 mA cm^−2^) levels to modulate the porosity of NAA in depth^33^. In our study, an apodization strategy was applied to the STPA profile with the aim of tuning the PSB of NAA-DBRs by engineering their effective medium in a stepwise fashion through different apodization windows. Four different apodization functions (i.e. linear positive, linear negative, logarithmic positive, and logarithmic negative) were used to modify the conventional STPA process. Furthermore, we systematically modified the amplitude difference (i.e. current density amplitude difference between the initial (*t*_0_) and half anodization time (*t*_*1/2*_) under ASTPA − *ΔA*_*J*_ = |*A*_*J*_ (*t*_0_) − *A*_*J*_ (*t*_*1/2*_)|), from *ΔA*_*J*_ = 0.105 mA cm^−2^ to 0.420 mA cm^−2^ with a step size of 0.105 mA cm^−2^. Figure 3 shows the ASTPA profiles used to produce apodized NAA-DBRs by each apodization function and amplitude difference. These profiles reveal that, under the anodization conditions used in our study (see Methods), modifications of the current density (input) are directly translated into voltage (output) changes, which is a critical factor for the accurate translation of current density profiles into porosity modulation in depth during anodization.

### Structural Characterization of NAA-DBRs

**Figure 4:**
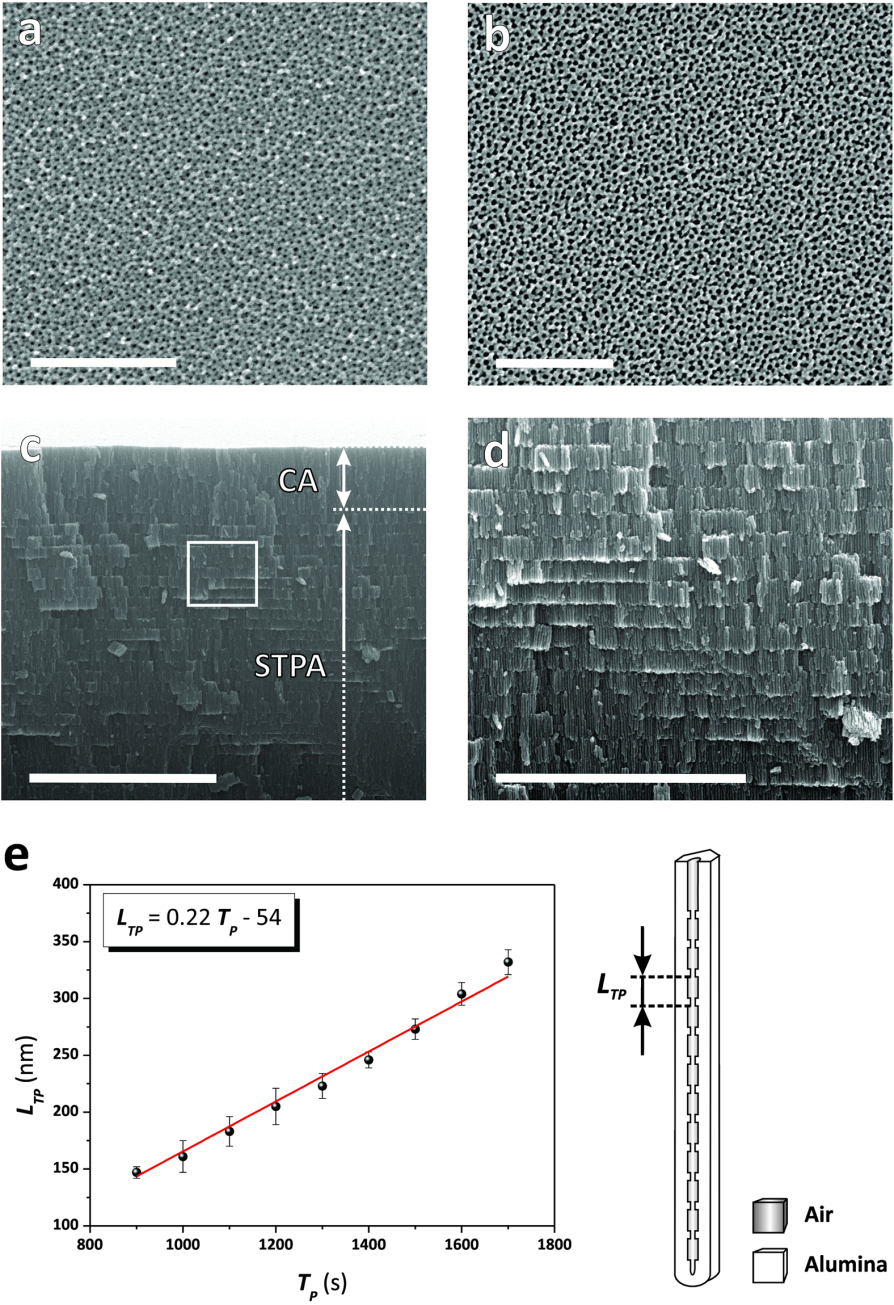
Representative FEG-SEM images of NAA-DBRs produced by STPA (Note; CA = constant current density anodization step). (**a**) Top view SEM image of a NAA-DBR produced with *T*_*P*_ = 1000 s, *A*_*J*_ = 0.420 mA cm^−2^, *J*_*Offset*_ = 0.280 mA cm^−2^, *t*_*STPA*_ = 20 h, and *t*_*pw*_ = 0 min (scale bar = 500 nm). (**b**) Top view SEM image of a NAA-DBR produced with *T*_*P*_ = 1000 s, *A*_*J*_ = 0.420 mA cm^−2^, *J*_*Offset*_ = 0.280 mA cm^−2^, *t*_*STPA*_ = 20 h, and *t*_*pw*_ = 6 min (scale bar = 500 nm). (**c**) General cross-sectional SEM image of a NAA-DBR showing the stacked layered structure with stepwise modulated porosity in depth (scale bar = 5 μm). (**d**) Magnified view of the white square shown in (**c**) (scale bar = 3 μm). (**e**) Linear correlation establishing the dependency of the period length (*L*_*TP*_) with the anodization period (*T*_*P*_) from *T*_*P*_ = 900 to 1700 s and schematic definition of *L*_*TP*_ in NAA-DBRs.

Figure 4 compiles a set of representative field-emission gun scanning electron microscopy (FEG-SEM) images of NAA-DBRs produced in our study. As these images reveal, NAA-DBRs feature an even but random distribution of nanopores across their surface, the average diameter (*d*_*p*_) of which was estimated to be *d*_*p*_ = 10 ± 3 and 19 ± 3 nm, for pore widening times (*t*_*pw*_) 0 and 6 min, respectively (Fig. 4a and b). This range of pore sizes was found to be optimal to obtain well-defined and intense PSBs in the transmission spectra of NAA-DBRs. NAA-based PC structures featuring bigger pore sizes, such as those produced in oxalic and phosphoric acids, scatter and absorb more light when photons travel across the PC structure, resulting in less intense PSBs. The resulting PCs feature a modulation of the pore diameter in depth that follows with precision the stepwise current density profile applied during STPA and ASTPA (Fig. 4c and d). This results in an in-depth modification of the effective refractive index of NAA that enables the generation of 1D NAA-DBRs. We also observed a linear relationship between the anodization period (*T*_*P*_) and the period length (*L*_*TP*_), defined as the distance between adjacent layers in the stacked structure of NAA-DBRs, where the latter changes at a rate of 0.22 nm s^−1^ with the former (Fig. 4e).

### Effect of Apodization on Optical Properties of NAA-DBRs

The transmission spectrum of NAA-DBRs displays a characteristically broad PSB with due to discontinuities in the effective refractive index profile^34^. Figure 1e shows the transmission spectra of a NAA-DBR produced with an anodization period of 1700 s. It is well-known that the PSB of DBRs can be engineered through apodization, optical filtering technique used to narrow the bandwidth of PC structures^35^. Figure 1f shows the transmission spectra of a NAA-DBR produced with *T*_*P*_ = 1700 s by a ASTPA profile apodized with a logarithmic negative function. It is clearly seen that the PSB of the NAA-DBR is significantly narrowed after apodizing the anodization profile. This effect is also observed for the different transmission orders of the PSB, where the apodized PC structure shows much well resolved and sharper PSBs than its non-apodized counterpart. Therefore, to apply an apodization approach to the STPA profile under the conditions used in our study enables the fine tuning of the light-filtering features of NAA-DBRs for specific applications, such as highly selective optical filters, high quality resonators, and ultrasensitive optical sensors.

**Figure 5:**
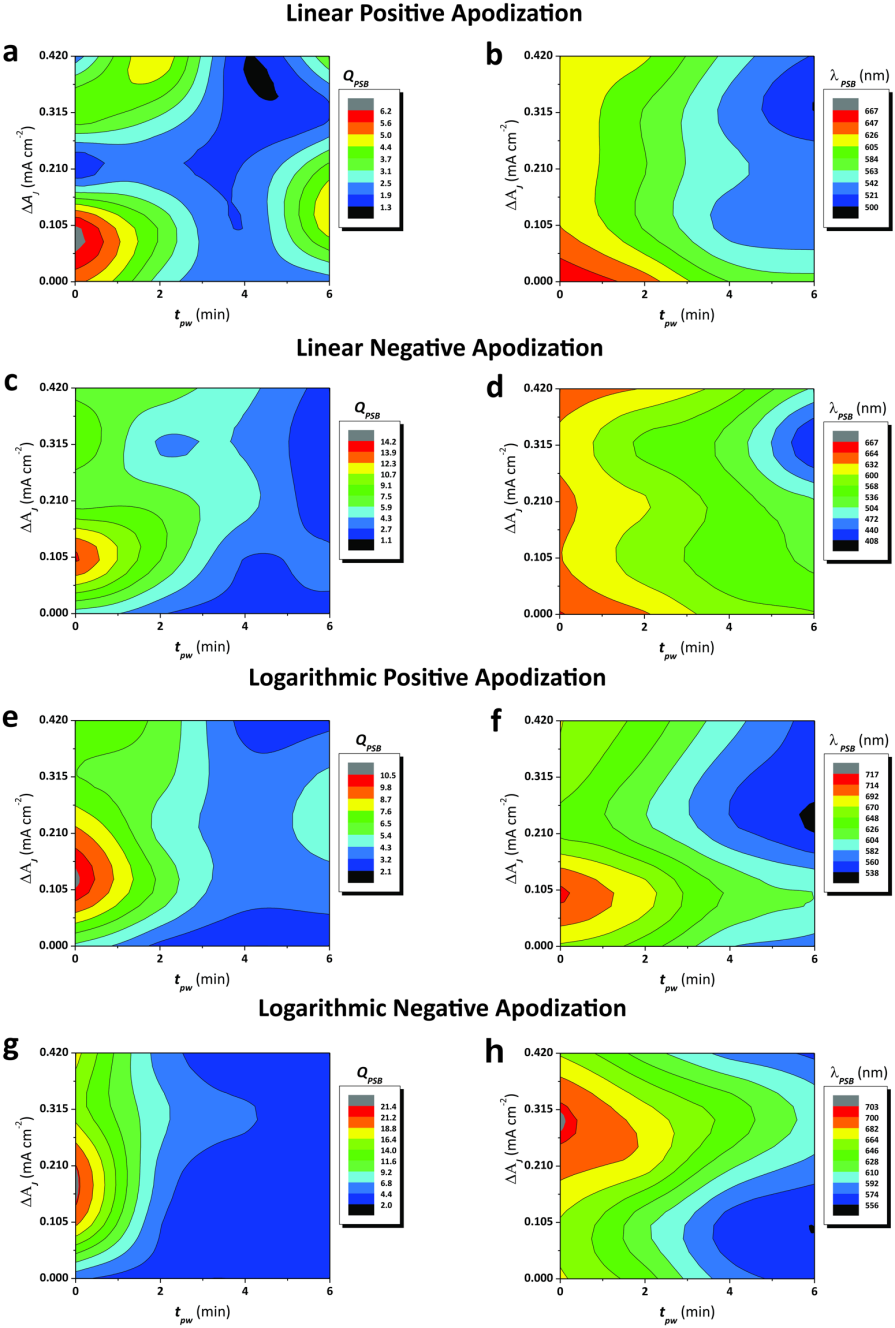
Contour maps showing the dependence of *Q*_*PSB*_ and *λ*_*PSB*_ of apodized NAA-DBRs as a function of the fabrication parameters (*ΔA*_*J*_ = *A*_*max*_ − *A*_*min*_ – amplitude difference and *t*_*pw*_ – pore widening time) (Note: *ΔA*_*J*_ = 0 – non-apodized NAA-DBRs). (**a** and **b**) Values of *Q*_*PSB*_ and *λ*_*PSB*_ as a function of *ΔA*_*J*_ and *t*_*pw*_ for linear positive apodization, respectively. (**c** and **d**) Values of *Q*_*PSB*_ and *λ*_*PSB*_ as a function of *ΔA*_*J*_ and *t*_*pw*_ for linear negative apodization, respectively. (**e** and **f**) Values of *Q*_*PSB*_ and *λ*_*PSB*_ as a function of *ΔA*_*J*_ and *t*_*pw*_ for logarithmic positive apodization, respectively. (**g** and **h**) Values of *Q*_*PSB*_ and *λ*_*PSB*_ as a function of *ΔA*_*J*_ and *t*_*pw*_ for logarithmic negative apodization, respectively.

Figure 5 shows contour maps summarizing the dependence of the central wavelength (*λ*_*PSB*_) and the quality factor (*Q*_*PSB*_ – calculated as the position of the central wavelength divided by the PSB’s full width at half maximum (*FWHM*_*PSB*_) – Equation 1) of the PSB of NAA-DBRs with the apodization function (i.e. linear positive, linear negative, logarithmic positive, and logarithmic negative), the amplitude difference (*ΔA*_*J*_), and the pore widening time (*t*_*pw*_). These graphs show how *Q*_*PSB*_ and *λ*_*PSB*_ vary with the fabrication parameters, enabling optimization paths to precisely tune the filtering features of NAA-DBRs.

1 $${Q}_{PSB}=\frac{{\lambda }_{PSB}}{FWH{M}_{PSB}}$$

Figure 5a and b show the effect of *ΔA*_*J*_ and *t*_*pw*_ on the filtering features of NAA-DBRs apodized following a linear positive function. Figure 5a denotes three maxima regions where *Q*_*PSB*_ presents the highest values, from *ΔA*_*J*_ = 0.210 to 0.420 mA cm^−2^ and *t*_*pw*_ = 0 to 2 min; from *ΔA*_*J*_ = 0.105 to 0.210 mA cm^−2^ and *t*_*pw*_ = 0 to 2 min; and from *ΔA*_*J*_ = 0.105 to 0.210 mA cm^−2^ and *t*_*pw*_ = 4 to 6 min. Linear positive apodized NAA-DBRs achieve the highest quality factor (6.2 ± 0.3) at *ΔA*_*J*_ = 0.105 mA cm^−2^ and *t*_*pw*_ = 0 min, as indicated by the red region in the contour plot. In contrast to *Q*_*PSB*_, *λ*_*PS*B_ was found to change smoothly with *ΔA*_*J*_ and *t*_*pw*_, as revealed by the homogeneous distance between color fields (Fig. 5b). *λ*_*PSB*_ undergoes a blue shift as *t*_*pw*_ and *ΔA*_*J*_ increase, although the magnitude of the blue shift produced by *t*_*pw*_ is more significant than that of *ΔA*_*J*_. Figure 5c and d display the effect of *ΔA*_*J*_ and *t*_*pw*_ on the central wavelength position and the quality factor of NAA-DBRs produced by a linear negative apodization function. It is observed that *Q*_*PSB*_ features a weak correlation with *ΔA*_*J*_ and *t*_*pw*_ at higher values of these two fabrication parameters (Fig. 5c). However, the quality factor of these NAA-DBRs increases significantly as *ΔA*_*J*_ and *t*_*pw*_ decrease, reaching its maximum value of 14.1 ± 0.7 at *ΔA*_*J*_ = 0.105 mA cm^−2^ and *t*_*pw*_ = 0 min. In the case of the position of the central wavelength (Fig. 5d), this is red-shifted as both *ΔA*_*J*_ and *t*_*pw*_ decrease. Likewise for a linear positive apodization, this change is smooth and homogeneous as denoted by the even distribution of color fields, although the effect of *ΔA*_*J*_ on *λ*_*PSB*_ is less significant than that of *t*_*pw*_. Figure 5e and f show the dependence of *Q*_*PSB*_ and *λ*_*PSB*_ with *ΔA*_*J*_ and *t*_*pw*_ for NAA-DBRs apodized with a logarithmic positive function. Figure 5e reveals that *Q*_*PSB*_ evolves homogeneously with *ΔA*_*J*_ and *t*_*pw*_. A reduction of the amplitude difference and pore widening time results in an enhancement of the quality factor of these NAA-DBRs, where *Q*_*PSB*_ is strongly dependent on these fabrication parameters as its value is nearby the maximum (*Q*_*PSB*_ = 10.5 ± 0.5), located at *ΔA*_*J*_ = 0.105 mA cm^−2^ and *t*_*pw*_ = 0 min. A similar trend is observed for the correlation between *λ*_*PSB*_ and *ΔA*_*J*_ and *t*_*pw*_, with a marked dependence on the fabrication parameters around its maximum (*λ*_*PSB*_ = 716 ± 1 nm), located at *ΔA*_*J*_ = 0.105 mA cm^−2^ and *t*_*pw*_ = 0 min. In good agreement with previous studies^36,37^, the position of the central wavelength is blue-shifted with *t*_*pw*_ following a linear fashion, as denoted by the equidistant separation between color fields as *t*_*pw*_ increases. Finally, Fig. 5g and h depict the relationship between *Q*_*PSB*_ and *λ*_*PSB*_ with *ΔA*_*J*_ and *t*_*pw*_ for NAA-DBRs produced by a logarithmic negative apodization approach. Figure 5g denotes a high concentration of color fields at short pore widening times, where the field lines are closer each other around the maximum. The combination of fabrication parameters that gives the highest *Q*_*PSB*_ (21.4 ± 1.0) for these NAA-DBRs is *ΔA*_*J*_ = 0.210 mA cm^−2^ and *t*_*pw*_ = 0 min. As far as *λ*_*PSB*_ is concerned, it is observed that the central wavelength undergoes a homogenous variation with *ΔA*_*J*_ and *t*_*pw*_, in a similar way than that of NAA-DBRs produced by a logarithmic positive apodization. However, the maximum value of *λ*_*PSB*_ is located at *ΔA*_*J*_ = 0.315 mA cm^−2^ and *t*_*pw*_ = 0 min.

**Figure 6:**
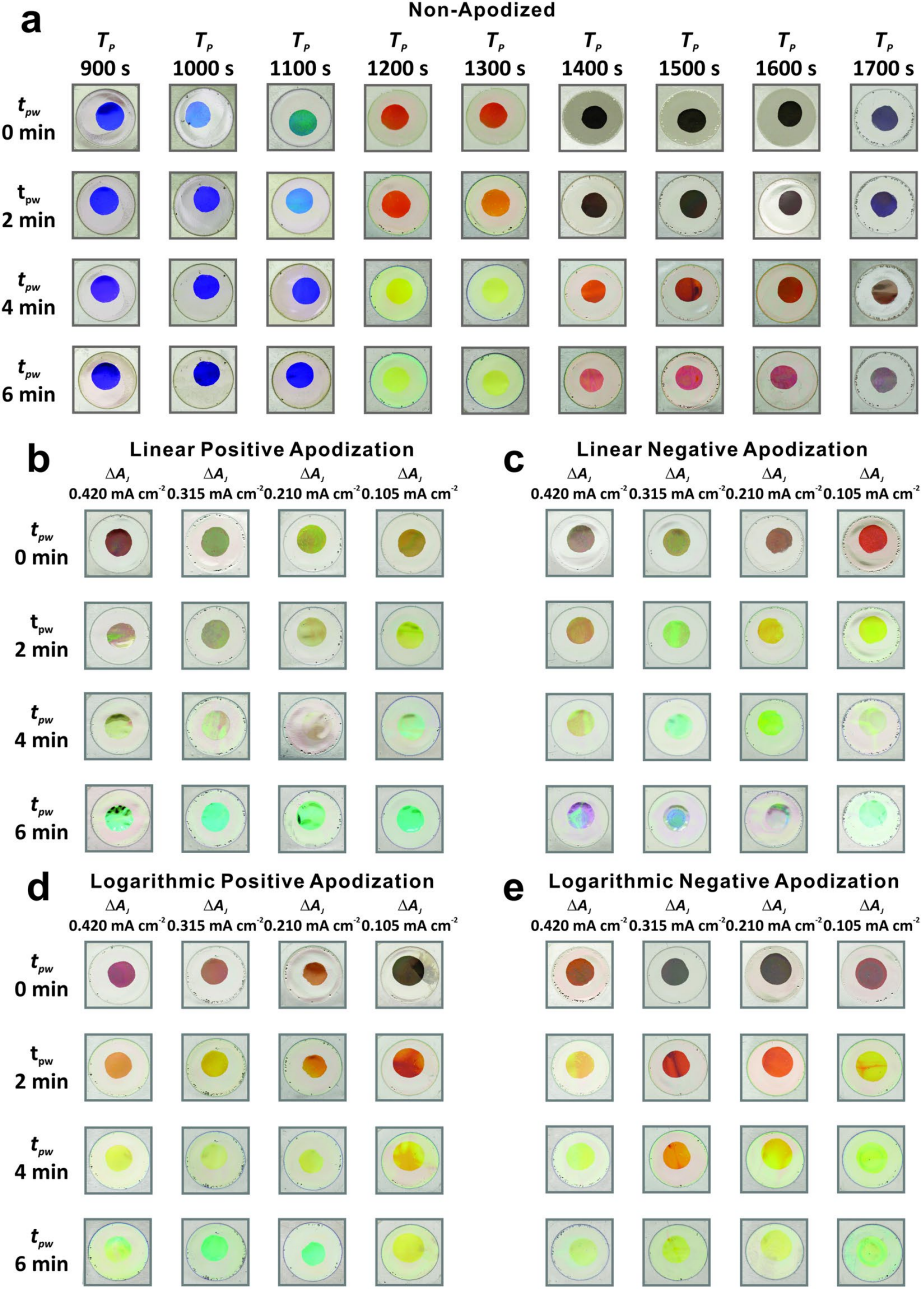
Digital pictures of non-apodized and apodized NAA-DBRs produced by STPA and ASTPA, respectively (diameter = 1 cm). (**a**) Non-apodized NAA-DBRs produced by STPA with *T*_*P*_ from 900 to 1700 s and *t*_*pw*_ from 0 min to 6 min (Note: fixed parameters – *A*_*J*_ = 0.420 mA cm^−2^, *J*_*Offset*_ = 0.280 mA cm^−2^, and *t*_*STPA*_ = 20 h). (**b**–**e**) Apodized NAA-DBRs produced by ASTPA with *ΔA*_*J*_ from 0.105 to 0.420 mA cm^−2^ and *t*_*pw*_ from 0 to 6 min for (**b**) linear positive apodization, (**c**) linear negative apodization, (**d**) logarithmic positive apodization, and (**e**) logarithmic negative apodization (Note: fixed parameters – *T*_*P*_ = 1300 s, *J*_*Offset*_ = 0.280 mA cm^−2^, and *t*_*ASTPA*_ = 20 h).

Another interesting property of NAA-DBRs is that these PC structures display vivid interferometric colors when the position of the central wavelength is located within the visible range of the spectrum. Figure 6a compiles a set of digital pictures of non-apodized NAA-DBRs produced at different anodization periods (*T*_*P*_ = 900 to 1700 s with *ΔT*_*P*_ = 100 s) and pore widening times (*t*_*pw*_ = 0 to 6 min with *Δt*_*pw*_ = 2 min). Figure 6b–e show digital pictures of apodized NAA-DBRs produced with an anodization period of 1300 s with four apodization functions (linear positive, linear negative, logarithmic positive, and logarithmic negative) as a function of *ΔA*_*J*_ and *t*_*pw*_. These pictures demonstrate that apodized and non-apodized NAA-DBRs display vivid colors such as orange, yellow, cyan and green, which corresponds to the position of their characteristic PSB within the visible range. This property can be readily engineered by modifying the fabrication parameters. For instance, linear negative apodized NAA-DBR with *ΔA*_*J*_ = 0.105 mA cm^−2^ and *t*_*pw*_ = 2 min displays yellow color, which corresponds to the position of its PSB at *λ*_*PSB*_ = 580 ± 1 nm. On the other hand, NAA-DBRs with their characteristic PSB in the NIR region (e.g. logarithmic negative apodized NAA-DBRs with *ΔA*_*J*_ = 0.315 mA cm^−2^ and *t*_*pw*_ = 0 min, where *λ*_*PSB*_ = 703 ± 1 nm) are transparent in color (black background).

To summarize, different apodization functions were explored to produce NAA-DBRs with optimized optical properties in terms of *Q*_*PSB*_ and *λ*_*PSB*_. All four apodization strategies (i.e. linear positive, linear negative, logarithmic positive and logarithmic negative) have shown to improve the quality of PSB as compared to non-apodized NAA-DBRs by certain combinations of fabrication parameters (i.e. *ΔA*_*J*_ and *t*_*pw*_). Among these functions, logarithmic negative apodization was found to be the most effective strategy to enhance the quality of the PSB of NAA-DBRs, as proven by the high value of *Q*_*PSB*_ (21.4 ± 1.0).

### Effect of Anodization Offset and Period on Optical Properties of NAA-DBRs

**Figure 7:**
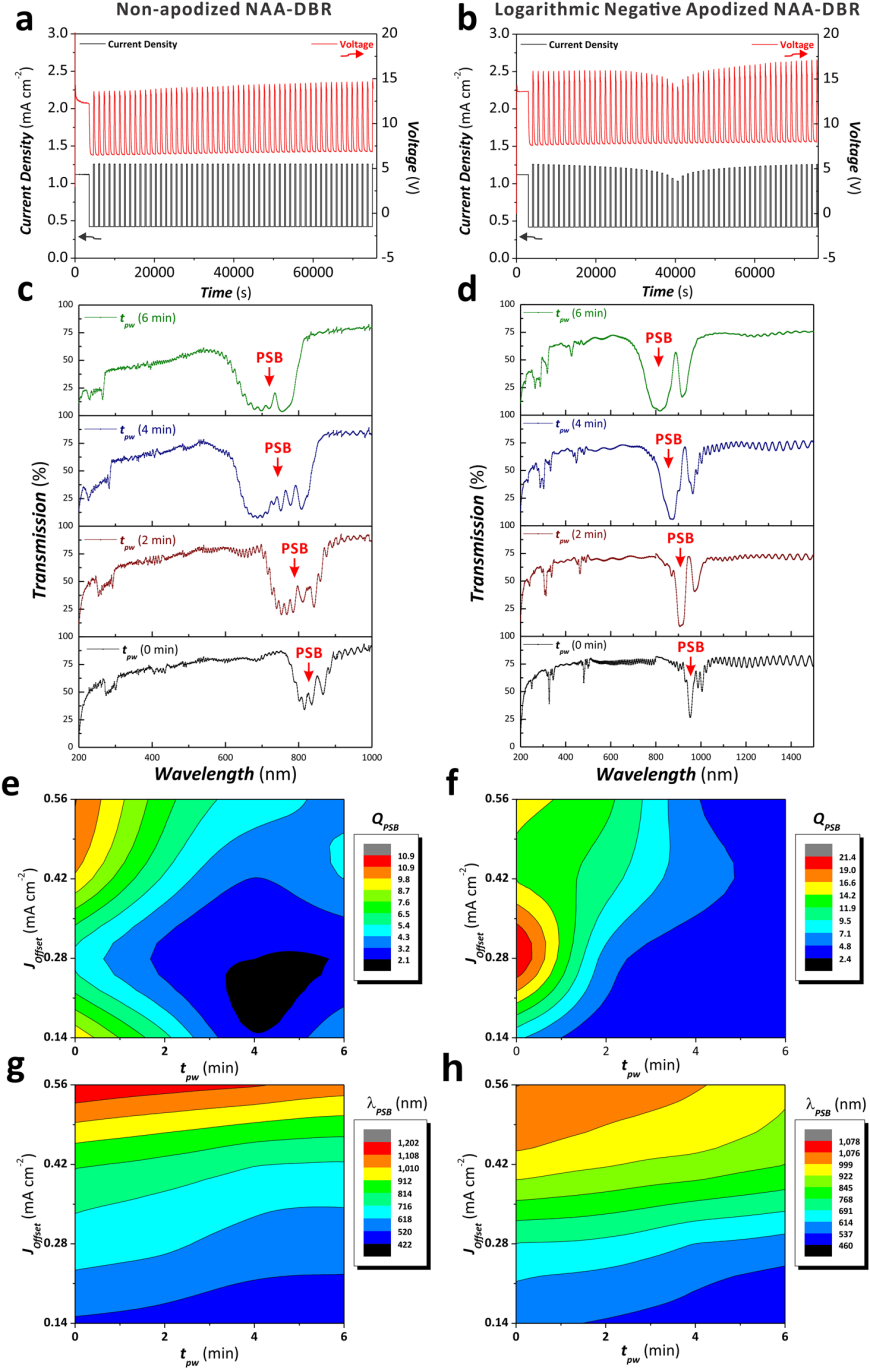
Effect of the current density offset (*J*_*Offset*_) and the pore widening time (*t*_*pw*_) on the *Q*_*PSB*_ and *λ*_*PSB*_ of non-apodized and logarithmic negative apodized NAA-DBRs. (**a** and **b**) Representative anodization profiles of non-apodized (*T*_*P*_ = 1500 s, *A*_*J*_ = 0.420 mA cm^−2^, and *t*_*STPA*_ = 20 h) and apodized NAA-DBRs (apodization function = logarithmic negative, *T*_*P*_ = 1500 s, *ΔA*_*J*_ = 0.210 mA cm^−2^, and *t*_*ASTPA*_ = 20 h), respectively. (**c** and **d**) Transmission spectra of non-apodized and apodized NAA-DBRs showing the PSB as a function of the pore widening time, respectively. (**e** and **f**) Contour maps showing the dependency of *Q*_*PSB*_ of non-apodized and apodized NAA-DBRs as a function of *J*_*Offset*_ and *t*_*pw*_, respectively. (**g** and **h**) Contour maps showing the dependency of *λ*_*PSB*_ of non-apodized and apodized NAA-DBRs as a function of *J*_*Offset*_ and *t*_*pw*_, respectively.

**Figure 8:**
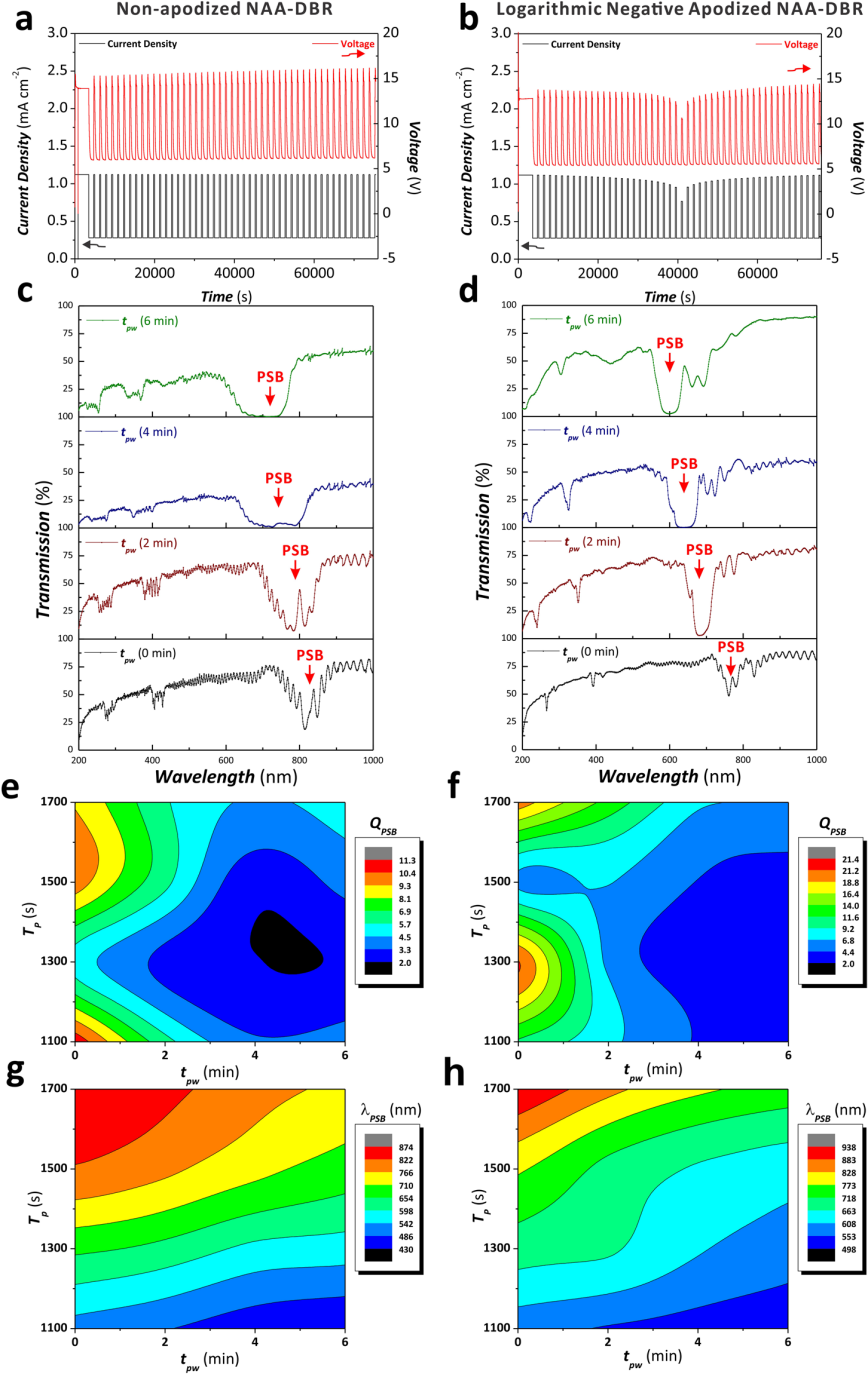
Effect of the anodization period (*T*_*P*_) and the pore widening time (*t*_*pw*_) on the *Q*_*PSB*_ and *λ*_*PSB*_ of non-apodized and apodized NAA-DBRs. (**a** and **b**) Representative anodization profiles of non-apodized (*T*_*P*_ = 1100 s, *A*_*J*_ = 0.420 mA cm^−2^, *J*_*min*_ = *J*_*offset*_ = 0.280 mA cm^−2^, and *t*_*STPA*_ = 20 h) and apodized NAA-DBRs (apodization function = logarithmic negative, *T*_*P*_ = 1100 s, Δ*A*_*J*_ = 0.210 mA cm^−2^, *J*_*min*_ = *J*_*offset*_ = 0.280 mA cm^−2^, and *t*_*ASTPA*_ = 20 h), respectively. (**c** and **d**) Transmission spectra of non-apodized and apodized NAA-DBRs showing the PSB as a function of the pore widening time, respectively. (**e** and **f**) Contour maps showing the dependency of *Q*_*PSB*_ of non-apodized and apodized NAA-DBRs as a function of *T*_*P*_ and *t*_*pw*_, respectively. (**g** and **h**) Contour maps showing the dependency of *λ*_*PSB*_ of non-apodized and apodized NAA-DBRs as a function of *T*_*P*_ and *t*_*pw*_, respectively.

To further optimize the optical properties of NAA-DBRs, the combined effect of the current density offset (*J*_*offset*_) and anodization period (*T*_*P*_) with *t*_*pw*_ on the optical properties of NAA-DBRs was systematically investigated by fabricating a set of non-apodized and apodized NAA-DBRs with different *J*_*offset*_ (from 0.140 to 0.560 mA cm^−2^ with *ΔJ*_*offset*_ = 0.140 mA cm^−2^) (Fig. 7) and *T*_*P*_ (from 1100 to 1700 s with *ΔT*_*P*_ = 200 s) (Fig. 8). Figure 7a and b show representative anodization profiles of logarithmic negative apodized (*J*_*offset*_ = 0.420 mA cm ^−2^, *T*_*P*_ = 1300 s, and *ΔA*_*J*_ = 0.210 mA cm^−2^) and non-apodized NAA-DBRs (*J*_*offset*_ = 0.420 mA cm ^−2^, *T*_*P*_ = 1300 s, and *A*_*J*_ = 0.420 mA cm^−2^) produced with different *J*_*Offset*_. Figure S1 (Supporting Information) compiles the anodization profiles of all the samples analyzed in our study. A qualitative comparison of the transmission spectra of these NAA-DBRs (Fig. 7c and d, respectively) reveals that a logarithmic negative apodization function significantly enhances the quality of the PSB of NAA-DBRs. Similarly to previous observations, *t*_*pw*_ intensifies and broadens the PSB in both apodized and non-apodized NAA-DBRs. The combined effect of *J*_*offset*_ and *t*_*pw*_ on the *Q*_*PSB*_ and *λ*_*PSB*_ of non-apodized and apodized NAA-DBRs is summarized in the contour maps shown in Fig. 7e and f, respectively. As Fig. 7e reveals, the color fields and field line distances are broad at longer *t*_*pw*_ and higher *J*_*offset*_ for non-apodized NAA-DBRs. The dependence of *Q*_*PSB*_ on *t*_*pw*_ increases as *t*_*pw*_ decreases, particularly at *J*_*offset*_ = 0.140 and 0.560 mA cm^−2^, as indicated by the denser field lines around the maximum (*Q*_*PSB*_ = 10.9 ± 0.5) located at *J*_*offset*_ = 0.560 mA cm^−2^ and *t*_*pw*_ = 0 min. Figure 7f displays the distribution of *Q*_*PSB*_ as a function of *J*_*offset*_ and *t*_*pw*_ for logarithmic negative apodized NAA-DBRs. The field lines from *J*_*offset*_ = 0.420 to 0.560 mA cm^−2^ and from *t*_*pw*_ = 2 to 6 min are wide apart, indicating that *Q*_*PSB*_ in apodized NAA-DBRs has a weak correlation with the pore widening time and the current density offset within the range of fabrication parameters assessed. The maximum of *Q*_*PSB*_ (21.4 ± 1.0) is achieved when *J*_*offset*_ and *t*_*pw*_ are set to 0.280 mA cm^−2^ and 0 min, respectively. The color field and field lines concentrate around this maximum, where the dependency of *Q*_*PSB*_ on *J*_*offset*_ and *t*_*pw*_ increases. Both non-apodized and apodized NAA-DBRs have poor quality of PSB when they are over-etched (i.e. long *t*_*pw*_), as revealed by the low values of *Q*_*PSB*_ and broad color fields within these regions of the contour maps. The distribution of *λ*_*PSB*_ with *J*_*offset*_ and *t*_*pw*_ for non-apodized and apodized NAA-DBRs is summarized in the contour maps shown in Fig. 7g and h, respectively. As these graphics reveal, *λ*_*PSB*_ of non-apodized NAA-DBRs has a stronger dependency with *J*_*offset*_ at high values of this fabrication parameter (from 0.420 to 0.560 mA cm^−2^) (Fig. 7g), while this dependence is stronger at moderate *J*_*offset*_ values in the case of apodized NAA-DBRs (from 0.280 to 0.420 mA cm^−2^) (Fig. 7h). However, in both cases it is observed a similar trend in the shift of *λ*_*PSB*_ with *J*_*offset*_ and *t*_*pw*_: when *J*_*offset*_ increases, *λ*_*PSB*_ is red-shifted toward the NIR region of the spectrum. In the case of *t*_*pw*_, the longer *t*_*pw*_ is the shorter the wavelength at which both non-apodized and apodized NAA-DBRs reflect light more efficiently (blue shift). The transmission spectra of all the NAA-DBRs analyzed in this study as a function of *J*_*offset*_ and *t*_*pw*_ are compiled in Figure S2 (Supporting Information).

Likewise in previous cases, these NAA-DBRs also display vibrant interferometric colors, which correspond to the position of the characteristic PSB across the UV-vis-NIR spectrum. Figure S3 (Supporting Information) displays digital pictures of non-apodized and logarithmic negative apodized NAA-DBRs produced at different values of *J*_*offset*_ and *t*_*pw*_. It is worthwhile to note that apodized NAA-DBRs have their characteristic PSB located at longer wavelengths as compared to their non-apodized counterparts. This is also denoted by the difference in the interferometric color displayed by these PCs. Furthermore, NAA-DBRs with high *J*_*offset*_ show transparent color as there is a red shift in the position of PSB (NIR region) as *J*_*offset*_ increases.

To assess the combined effect of the anodization period (*T*_*P*_) and *t*_*pw*_ on the optical filtering features of non-apodized and logarithmic negative apodized NAA-DBRs, *T*_*P*_ and *t*_*pw*_ were systematically modified from 1100 to 1700 s with *ΔT*_*P*_ = 200 s, and from 0 to 6 min with *Δt*_*pw*_ = 2 min, respectively. The obtained results showing the dependency of *Q*_*PSB*_ and *λ*_*PSB*_ with *T*_*P*_ and *t*_*pw*_ for these NAA-DBRs are summarized in Fig. 8. Figure 8a and b display representative anodization profiles of non-apodized and apodized NAA-DBRs produced with *T*_*P*_ = 1500 s, respectively, whereas Figure S4 (Supporting Information) compiles the anodization profiles of all the NAA-DBRs analyzed in this study. Representative transmission spectra of non-apodized (*T*_*P*_ = 1500 s, *A*_*J*_ = 0.420 mA cm^−2^, and *J*_*offset*_ = 0.280 mA cm^−2^) and logarithmic negative apodized (*T*_*P*_ = 1500 s, *ΔA*_*J*_ = 0.210 mA cm^−2^, and *J*_*offset*_ = 0.280 mA cm^−2^) NAA-DBRs at different *t*_*pw*_ are presented in Fig. 8c and d, respectively.

These graphs show that the transmission spectrum of apodized NAA-DBRs features much narrower PSBs than those of non-apodized NAA-DBRs. It is also observed that to widen the nanoporous structure of NAA-DBRs increases the intensity and width of the PSBs, independently on the anodization period (Figure S5 – Supporting Information).

Figure 8e and f show contour maps describing how *Q*_*PSB*_ varies with *T*_*P*_ and *t*_*pw*_ for non-apodized NAA-DBRs and logarithmic negative apodized NAA-DBRs, respectively. Both graphs exhibit similar *Q*_*PSB*_ distribution with broad color fields and field lines at longer *t*_*pw*_ and increasing concentration of color fields as *t*_*pw*_ decreases. Non-apodized NAA-DBRs display two maxima located at *T*_*P*_ = 1100 and 1500 s with *t*_*pw*_ = 0 min, where the former has the maximum value for *Q*_*PSB*_ (11.2 ± 0.6). In the case of apodized NAA-DBRs, the maxima are located at *T*_*P*_ = 1300 and 1700 s with *t*_*pw*_ = 0 min, where the former gives the maximum *Q*_*PSB*_ value of 21.3 ± 0.6, which is almost twice higher than that of the non-apodized counterparts. The dependency of *Q*_*PSB*_ with *T*_*P*_ increases around the maxima, as indicated by the close field lines and the concentration of color fields around these points. It is worth nothing that the *Q*_*PSB*_ of apodized NAA-DBRs has a stronger dependency on *T*_*P*_ as compared to non-apodized NAA-DBRs, denoted by the smaller field line distances in Fig. 8f.

The dependency of *λ*_*PSB*_ on *T*_*P*_ and *t*_*pw*_ for non-apodized and apodized NAA-DBRs is summarized in Fig. 8g and h, respectively. As shown by the contour map for non-apodized NAA-DBRs (Fig. 8g), the color fields are distributed homogenously throughout the map with equidistant field lines at *T*_*P*_ < 1500 s, which suggest a fairly strong dependence of *λ*_*PSB*_ on this range of *T*_*P*_. The distance between field lines increases from *T*_*P*_ = 1500 to 1700 s, indicating a weaker dependence of *λ*_*PSB*_ on *T*_*P*_. In contrast, the contour map for apodized NAA-DBRs (Fig. 8h) shows an even distribution of color fields with equidistant field lines at *T*_*P*_ > 1500 s and broader color fields with more distant field lines at *T*_*P*_ < 1500 s. These results indicates that the dependency of *λ*_*PSB*_ on *T*_*P*_ is stronger at longer *T*_*P*_ than that at shorter *T*_*P*_. Compared to *T*_*P*_, *t*_*pw*_ has a less significant impact on the distribution of *λ*_*PSB*_, with a similar effect in both non-apodized and apodized NAA-DBRs, where an increment in *T*_*P*_ red-shifts the position of *λ*_*PSB*_ while an increment in *t*_*pw*_ blue-shifts the position of *λ*_*PSB*_. This result is good agreement with previous studies, establishing the behavior of the characteristic PSB of NAA-based photonic structures under the manipulation of *T*_*P*_ and *t*_*pw*_^38^.

NAA-DBRs also displayed tunable vivid interferometric colors as the position of the PSB is shifted across spectral regions (Figure S6 **–** Supporting Information). The difference in the interferometric colors shown by non-apodized and apodized NAA-DBRs is due to the shift in the PSB’s position, which is located at longer wavelengths for apodized NAA-DBRs as compared to that of non-apodized NAA-DBRs produced with the same *T*_*P*_.

### Assessment of Effective Medium Sensitivity of Non-apodized and Apodized NAA-PCs

As revealed by the analysis of *Q*_*PSB*_, the implementation of an apodization strategy during anodization is an effective approach to improve the quality of the PSB of NAA-DBRs, which results in better resolved and narrower characteristic PSBs with tunable filtering features across the spectral regions. This feature can be readily used to develop highly sensitive optical sensing platforms to detect analytes of interest based on effective refractive index changes^17^. It is known that PC structures with high quality PSBs are more sensitive to effective medium changes (i.e. larger shifts in the position of PSB upon small effective medium changes), making them optimal platforms to develop advanced sensing system^39^.

**Figure 9:**
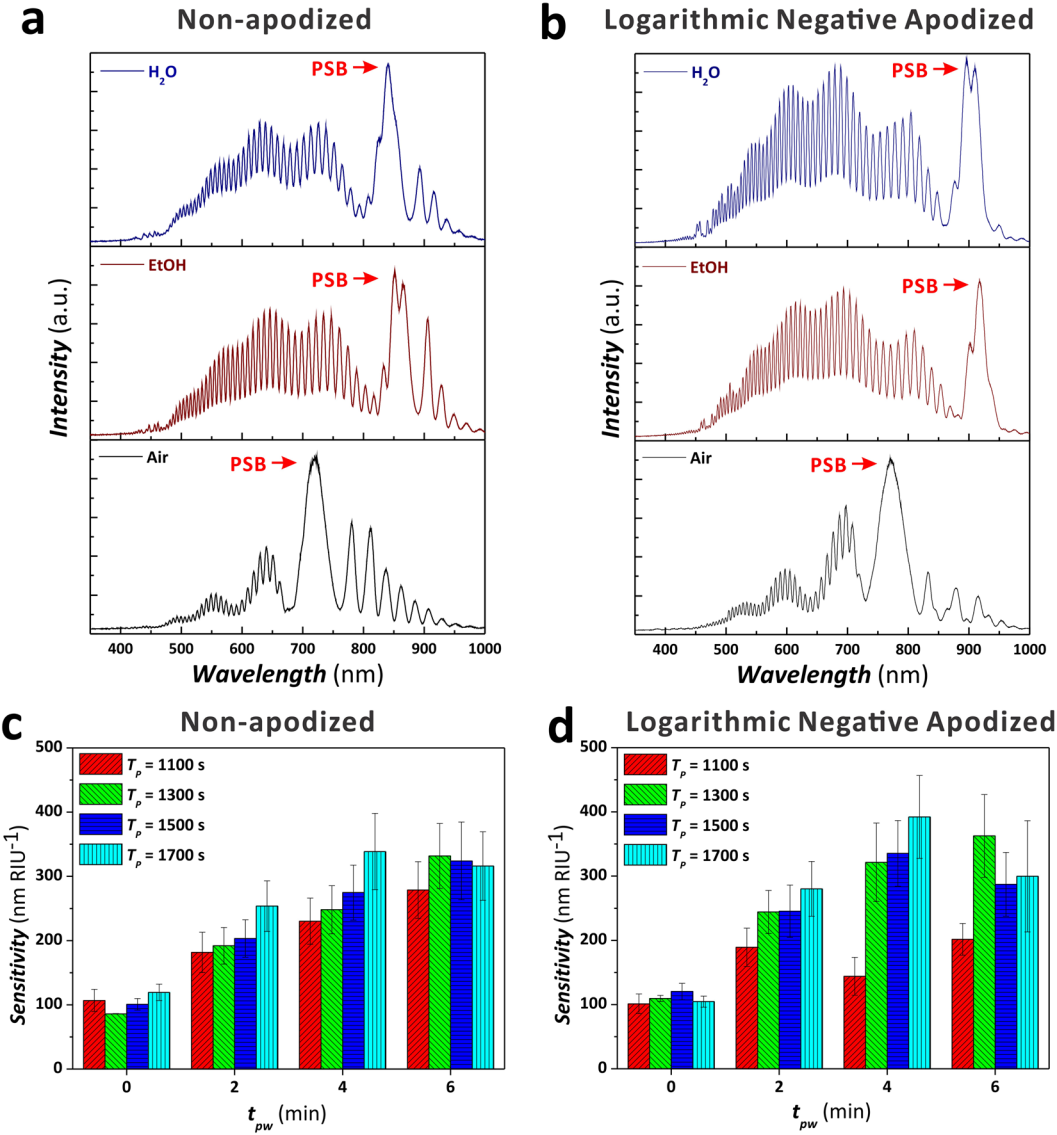
Assessment of sensitivity of NAA-DBRs upon effective medium changes in non-apodized and apodized NAA-DBRs. (**a**) Representative RIfS spectra of a non-apodized NAA-DBR under different nanopores-infiltrating medium (i.e. air, ethanol, and water) (*T*_*P*_ = 1700 s, *A*_*J*_ = 0.420 mA cm^−2^, *J*_*min*_ = *J*_*offset*_ = 0.280 mA cm^−2^, *t*_*STPA*_ = 20 h, and *t*_*pw*_ = 4 min). (**b**) Representative RIfS spectra of a logarithmic negative apodized NAA-DBR under different nanopores-infiltrating medium (i.e. air, ethanol, and water) (*T*_*P*_ = 1700 s, *ΔA*_*J*_ = 0.210 mA cm^−2^, *J*_*min*_ = *J*_*offset*_ = 0.280 mA cm^−2^, *t*_*ASTPA*_ = 20 h, and *t*_*pw*_ = 4 min). (**c**) Bar chart showing the sensitivity in nm RIU^−1^ of non-apodized NAA-DBRs produced at different *T*_*P*_ (1100, 1300, 1500, and 1700 s) and *t*_*pw*_ (0, 2, 4, and 6 min) (*A*_*J*_ = 0.420 mA cm^−2^, *J*_*min*_ = *J*_*offset*_ = 0.280 mA cm^−2^, and *t*_*STPA*_ = 20 h). (**d**) Bar chart showing the sensitivity in nm RIU^−1^ of logarithmic negative apodized NAA-DBRs produced at different *T*_*P*_ (1100, 1300, 1500, and 1700 s) and *t*_*pw*_ (0, 2, 4, and 6 min) (*ΔA*_*J*_ = 0.210 mA cm^−2^, *J*_*min*_ = *J*_*offset*_ = 0.280 mA cm^−2^, and *t*_*ASTPA*_ = 20 h).

To verify that apodized NAA-DBRs with better *Q*_*PSB*_ are more sensitive toward effective medium changes, we systematically infiltrated the nanoporous network of non-apodized and apodized NAA-DBRs produced with different *T*_*P*_ (i.e. 1100, 1300, 1500, and 1700 s) with media of different refractive index and measured shifts in the PSB (*Δλ*_*PSB*_) using reflectometric interference spectroscopy (RIfS). Figure 9a and b show representative RIfS spectra for non-apodized (*T*_*P*_ = 1700 s, *A*_*J*_ = 0.420 mA cm^−2^, *J*_*offset*_ = 0.280 mA cm^−2^, and *t*_*pw*_ = 4 min) and apodized NAA-DBRs (*T*_*P*_ = 1700 s, *ΔA*_*J*_ = 0.210 mA cm^−2^, *J*_*offset*_ = 0.280 mA cm^−2^, and *t*_*pw*_ = 4 min), respectively, infiltrated with different media (air ~1.00 RIU, water ~1.33 RIU, and ethanol ~1.36 RIU). As demonstrated in our previous study^22^, the RIfS spectra of NAA-DBRs features two main regions: namely; i) the interference of light from all layers of the multilayered NAA-DBR structure characterized by a relatively narrow and intense PSB, and ii) the Fabry-Pérot interference spectrum (i.e. fast oscillations) produced by the reflections of light at the interfaces bordering the NAA-DBR. In this study, we used the positon of the characteristic PSB in the RIfS spectra of NAA-DBRs as sensing parameter. The sensitivity of these PC structures as a function of *T*_*P*_ and *t*_*pw*_ is established by the slope of the linear fitting obtained by correlating *Δλ*_*PSB*_, determined by RIfS, and the refractive index of the medium filling the nanopores. The obtained results for non-apodized and apodized NAA-DBRs are summarized in Fig. 9c and d.

Figure 9c reveals that non-apodized NAA-DBRs produced with *T*_*P*_ = 1700 s have the highest sensitivity at *t*_*pw*_ = 0, 2, and 4 min (i.e. 119 ± 13, 254 ± 39, and 339 ± 60 nm RIU^−1^, respectively), whereas at *t*_*pw*_ = 6 min, those NAA-DBRs produced at *T*_*P*_ = 1300 s display the highest sensitivity (i.e. 332 ± 51 nm RIU^−1^, respectively). In contrast, Fig. 9d denotes that logarithmic negative apodized NAA-DBRs produced with *T*_*P*_ = 1500 s have the highest sensitivity at *t*_*pw*_ = 0 (120 ± 13 nm RIU^−1^, respectively). However, at *t*_*pw*_ = 2 and 4 min, apodized NAA-DBRs produced at *T*_*P*_ = 1700 s have the highest sensitivity (280 ± 43 and 392 ± 65 nm RIU^−1^, respectively) and, at *t*_*pw*_ = 6 min, NAA-DBRs fabricated with *T*_*P*_ = 1300 s are the most sensitive PC structures (363 ± 64 nm RIU^−1^).

In general, it is observed that the sensitivity of non-apodized and apodized NAA-DBRs is enhanced by the pore widening treatment for 0 min ≤ *t*_*pw*_ ≤ 4 min. However, as denoted by the *Q*_*PSB*_ analysis, further increase in *t*_*pw*_ worsens the quality of the PSB due to the over-etching of the nanoporous structure of NAA-DBRs, which broadens the PSB and reduces the sensitivity of these platforms. Interestingly, both non-apodized and apodized NAA-DBRs have exhibited the highest sensitivity (i.e. 339 ± 59 and 392 ± 65 nm RIU^−1^, respectively) at *t*_*pw*_ = 4 min and *T*_*P*_ = 1700 s, where the latter is revealed to be the most sensitive platform toward changes in the effective medium. The sensitivity of apodized NAA-DBRs was found to be ~ 16% higher than that of their non-apodized counterparts.

To conclude, this study provides new insights into the capability of anodization to engineer and tune the optical properties of NAA-based photonic crystal structures. An apodization strategy applied during stepwise pulse anodization enables the precise control over the features of the photonic stopband of NAA-DBRs by manipulating various anodization parameters (i.e. apodization function, amplitude difference, current density offset, anodisation period, and pore widening time). The systematic analysis on the effect of each fabrication parameter reveals that a logarithmic negative apodization function provides the highest quality of the photonic stopband (*Q*_*PSB*_ = 21.4 ± 1.0). Apodized NAA-DBRs are demonstrated to be a more sensitive sensing platform than non-apodized NAA-DBRs as they have shown higher sensitivity toward effective medium changes (~16% enhancement). Logarithmic negative apodized and non-apodized NAA-DBRs produced with *T*_*P*_ = 1700 s and *t*_*pw*_ = 4 min have a sensitivity of 392 ± 65 and 339 ± 59 nm RIU^−1^, respectively.

These innovative NAA-based photonic crystal structures with enhanced optical properties pave the way for the development of ultrasensitive optical sensing systems and other photonic elements such as selective optical filters with broad applicability.

## Methods

### Materials

High purity (99.9997%) aluminum (Al) foils of thickness 0.32 mm were supplied by Goodfellow Cambridge Ltd. (UK). Sulfuric acid (H_2_SO_4_), perchloric acid (HClO_4_), copper (II) chloride (CuCl_2_), hydrochloric acid (HCl), phosphoric acid (H_3_PO_4_), and ethanol (EtOH – C_2_H_5_OH) were supplied by Sigma-Aldrich (Australia) and used as received, without further purification. Aqueous solutions used in this study were prepared with ultrapure Mili-Q® water (18.2 mΩ.cm) (Australia).

### Fabrication of NAA Photonic Structures

Non-apodized NAA photonic structures were produced by stepwise pulse anodisation (STPA) approach under current density control conditions. 1.5 × 1.5 cm Al square chips were first washed in ethanol and water under sonification for 15 min each and dried under air stream. These Al substrates were then electropolished in a mixture of EtOH and HClO_4_ 4:1 *(v:v)* at 20 V and 5 °C for 3 min. After electropolishing, Al chips were anodized in an aqueous solution of 1.1 M H_2_SO_4_ with 25 *v*% of EtOH at −1 °C. The galvanostatic anodization process started with a constant step at a current density of 1.120 mA cm^−2^ for 1 h to achieve a homogenous pore growth rate prior to stepwise pulse anodization. The anodization profile was subsequently switched to stepwise pulse mode, where the current density was pulsed between high (*J*_*max*_ = 1.120 mA cm^−2^) and low (*J*_*min*_ = 0.280 mA cm^−2^) current density values following a stepwise modulation for a total anodization time (*t*_*STPA*_ or *t*_*ASTPA*_) of 20 h. A set of reference non-apodized NAA-DBRs were produced, where the current density amplitude (*A*_*J*_) and current density offset (*J*_*offset*_) were set at 0.420 mA cm^−2^ and 0.280 mA cm^−2^, respectively, whereas the anodization period (*T*_*P*_) (i.e. time between consecutive pulses) was modified from 900 to 1700 s with a step size of 100 s. Note that *T*_*P*_ is given by Equation 2.

2 $${T}_{P}={t}_{max}+{t}_{min}$$

where *t*_*max*_ and *t*_*min*_ are the time lengths at *J*_*max*_ and *J*_*min*_, respectively, and *t*_*max*_ and *t*_*min*_ were set at a ratio of 1:4 (i.e. *t*_*min*_ = 4*t*_*max*_) for *T*_*P*_ = 900 to 1700 s.

To assess the effect of *J*_*Offset*_, another set of non-apodized NAA photonic structures was fabricated based on the following parameters: *A*_*J*_ = 0.420 mA cm^−2^, *T*_*P*_ = 1300 s, where *J*_*offset*_ was adjusted from 0.140 to 0.560 mA cm^−2^ with an interval of 0.140 mA cm^−2^. The current density during stepwise pulse mode was pulsed between *J*_*min*_ and *J*_*max*_, where the these parameters were defined by Equations 3 and 4, respectively.

3 $${J}_{Low}={J}_{offset}$$

4 $${J}_{High}=2{A}_{J}+{J}_{offset}$$

### Apodization and Optical Tuning of the PSB of NAA-DBRs

Four different apodization functions: (i) linear positive, (ii) linear negative, (iii) logarithmic positive, and (iv) logarithmic negative, were implemented in a reference STPA profile (i.e. *T*_*P*_ = 1300 s, *J*_*min*_ = *J*_*offset*_ = 0.280 mA cm^−2^, *J*_*High*_ = 1.120 mA cm^−2^, *A*_*J*_ = 0.420 mA cm^−2^, and *t*_*STPA*_ = 20 h). The mathematical expressions for each apodization function are listed in Equations S1–S8 (Supporting Information). These ASTPA anodisation profiles were produced by a custom-designed Labview®-based software based on Equation 5:

5 $$J(t)=2{A}_{J}(t)+{J}_{offset}$$

where *A*_*J*_ *(t)* is the time-dependent current density amplitude defined for a time between a minimum (*A*_*min*_) and maximum (*A*_*max*_) of amplitude for the corresponding apodization functions (Equations S1–S8 – Supporting Information).

To investigate the effect of *ΔA*_*J*_ on the optical properties of NAA-DBRs, the amplitude difference (*ΔA*_*J*_) was modified from 0.105 to 0.420 mA cm^−2^ with an interval of 0.105 mA cm^−2^ for each apodization function. Using the most effective apodization function with optimized *ΔA*_*J*_, which was determined by analyzing the quality factor (*Q*_*PSB*_), other apodization parameters such as *J*_*Offset*_ and *T*_*P*_ were systematically modified from 0.140 to 0.560 mA cm^−2^ with a step size of 0.140 cm^−2^, and from 1100 to 1700 s with a step size of 200 s, respectively, in order to further optimize the optical signals as well as to tune the optical properties of NAA photonic structures.

### Optical Characterization

Prior to optical characterization, NAA-DBRs were etched chemically in a saturated solution of HCl/CuCl_2_ to dissolve the remaining aluminum substrate from the backside. This process was carried out using an etching mask with a circular window of 5 mm in diameter in an etching cell. The optical properties of these etched NAA photonic structures were characterized by analyzing their transmission spectra measured using a UV-visible-NIR spectrophotometer (Cary 300 and Cary 5000, Agilent, USA, for wavelength range of 200–1000 nm and 200–1500 nm respectively). These spectra were obtained at normal incidence (i.e. *θ* = 0°) with a resolution of 1 nm. To characterize the interferometric color exhibited by these NAA photonic structures, digital images with a black card as background were acquired using a Canon EOS 700D digital camera equipped with a Tamron 90 mm F2.8 VC USD macro mount lens with autofocus function under natural illumination. These NAA photonic crystals were pore widened using an aqueous solution of 5 *wt*% H_3_PO_4_ at 35 °C for *t*_*pw*_ from 0 to 6 min with an interval of 2 min. After each pore widening step, the optical characterization of NAA-DBRs was carried out by recording the transmission spectra and digital images as outlined above.

### Assessment of Sensitivity of Non-apodized and Apodized NAA-DBRs

The sensitivity of non-apodized and apodized NAA-DBRs was assessed by correlating the shift in the position of central wavelength (*λ*_*PSB*_) to the refractive index values of the medium filling the nanopores. The spectra of NAA-DBRs with medium-filled nanopores was measured and recorded using a reflectometric interference spectroscopy (RIfS) set-up. White light from a tungsten source (LS-1LL, Ocean Optics, USA) was focused on the surface of NAA-DBRs with an illumination spot of ~2 mm in diameter by a bifurcated optical probe. The collection fiber in the optical probe collected and transferred the light reflected from the illumination spot to a miniature spectrometer (USB 4000+VIS-NIR-ES, Ocean Optics, USA). The optical spectra acquired in the range of 400–1000 nm wavelength were saved with an integration time of 10 ms and 10 average measurements. Note that NAA-DBRs were coated with an ultra-thin layer of gold by a sputter coater (Sputter coater 108, Cressington, USA) to enhance intereferometric reflection. After recording the optical spectra of NAA-DBRs with air filling the nanopores, a drop of EtOH was placed on the surface and allowed to settle into the nanopores for a few seconds to ensure that the nanopores were infiltrated by EtOH. The spectrum was recorded once the signals were stabilized. Then, a drop of water was placed on the surface of NAA photonic crystals after EtOH had completely evaporated. The RIfS spectrum of NAA-DBRs with H_2_O-filled nanopores was recorded and saved. NAA photonic crystals measured were pore widened for *t*_*pw*_ = 0 to 6 min. After each pore widening step, RIfS spectra were recorded for each NAA-DBRs with different medium filling the nanopores (i.e. air, EtOH, and H_2_O). The spectra acquired were then processed using Igor Pro library (Wavemetrics, USA) to determine *Δλ*_*PSB*_ in non-apodized and apodized NAA-DBRs.

### Structural Characterization of NAA-DBRs

The morphology and structure of NAA photonic crystals were characterized by FEG-SEM image analysis from images acquired by field emission gun scanning electron microscopy (FEG-SEM FEI Quanta 450). The acquired FEG-SEM images were analyzed using ImageJ (public domain program developed at RSB of the NIH)^40^.

## Electronic supplementary material

Supplementary Information
