# Supplementary material for: On the Precise Tuning of Optical Filtering Features in Nanoporous Anodic Alumina Distributed Bragg Reflectors
Source: Sci Rep. 2018 Mar 15;8:4642. doi: 10.1038/s41598-018-22895-5 (PMC5854698; doi:10.1038/s41598-018-22895-5)
Supplement: Supplementary file 1 — Supplementary Information [file 41598_2018_22895_MOESM1_ESM.pdf]

Supporting Information

On the Precise Tuning of Optical Filtering Features in Nanoporous Anodic Alumina Distributed Bragg Reflectors

Cheryl Suwen Law[1,2,3], Siew Yee Lim[1,2,3], and Abel Santos[1,2,3*]

[1]School of Chemical Engineering, The University of Adelaide, Adelaide, SA 5005, Australia
[2]Institute for Photonics and Advanced Sensing (IPAS), The University of Adelaide, 5005 Adelaide, Australia
[3]ARC Centre of Excellence for Nanoscale BioPhotonics (CNBP), The University of Adelaide, 5005 Adelaide, Australia

***E-Mail:** abel.santos@adelaide.edu.au

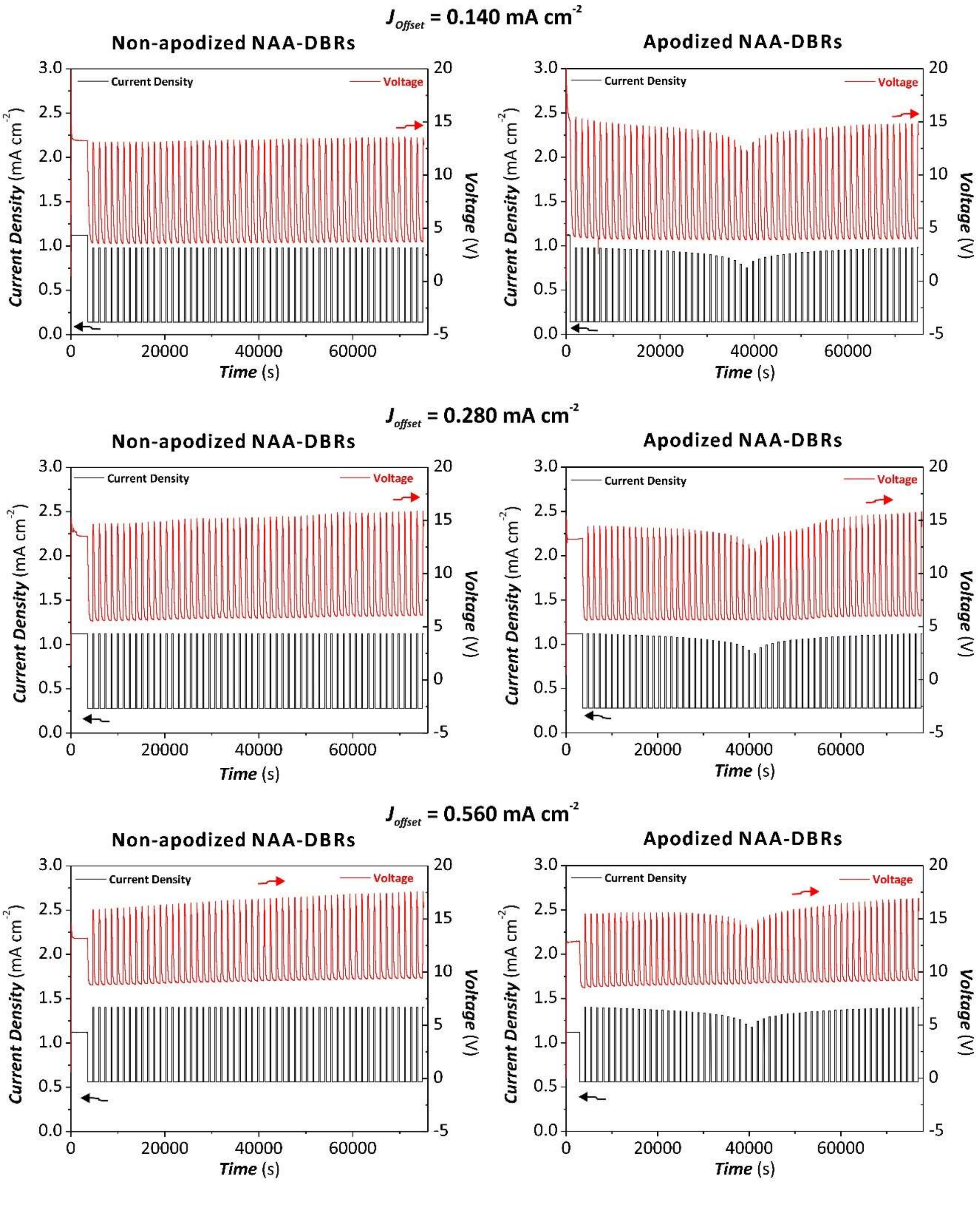

Figure S1. Anodization profiles of non-apodized and logarithmic negative apodized NAA-DBRs as a function of J_{Offset}.

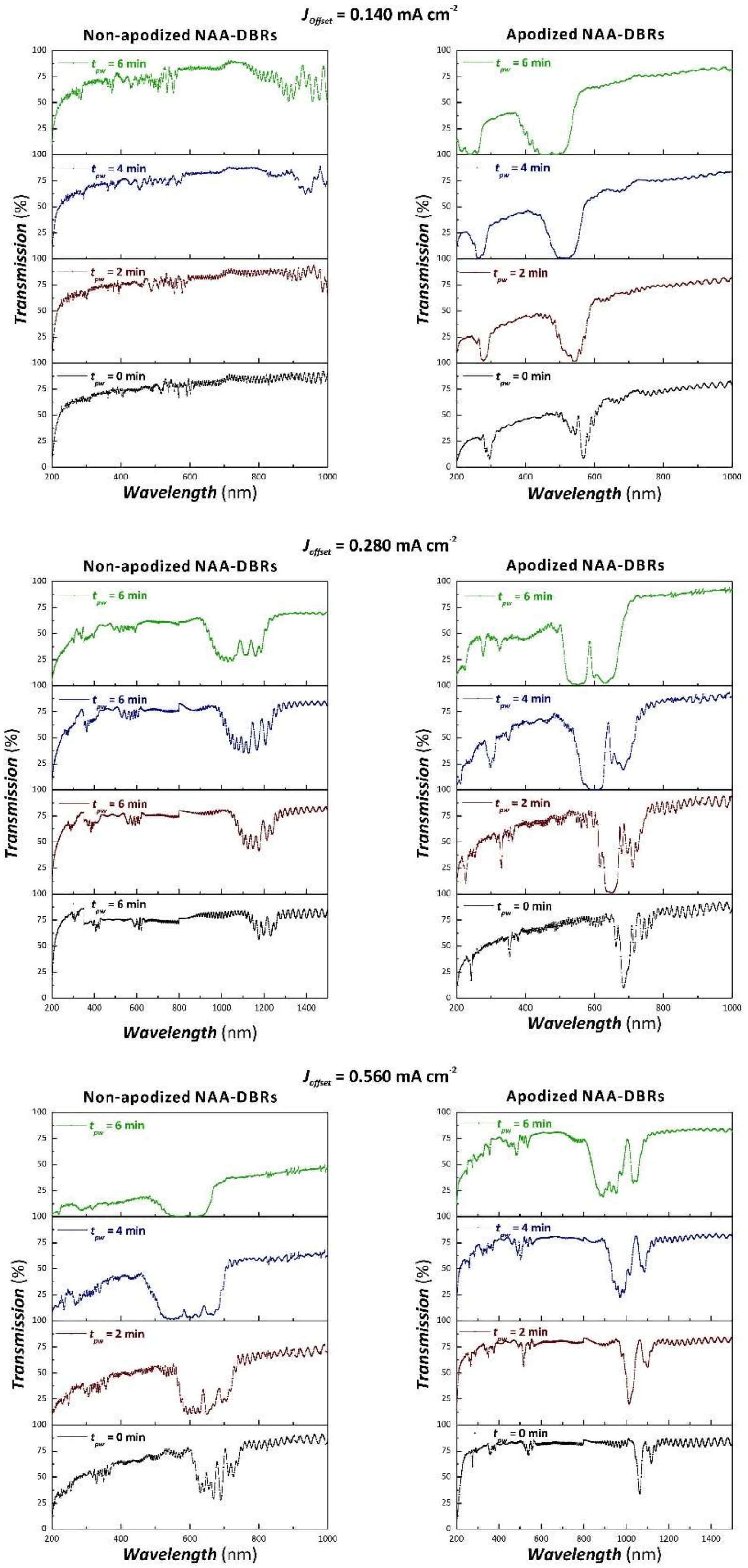

Figure S2. Transmission spectra of non-apodized and logarithmic negative apodized NAA-DBRs as a function of J_{Offset} and t_{pw}.

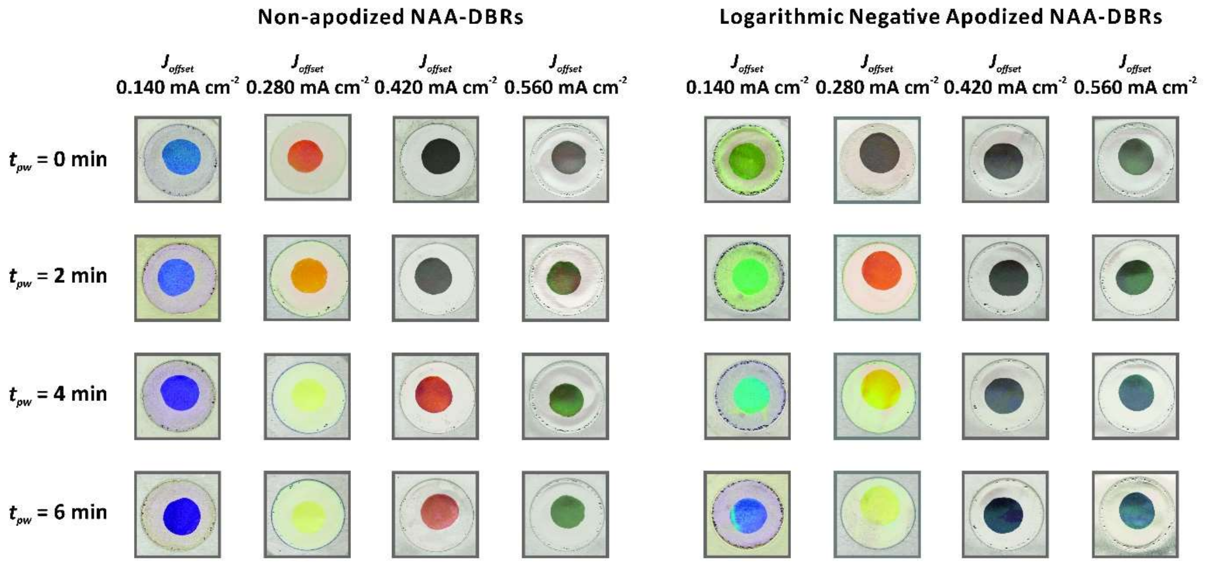

Figure S3. Digital pictures of non-apodized and logarithmic negative apodized NAA-DBRs as a function of J_{Offset} and t_{pw}.

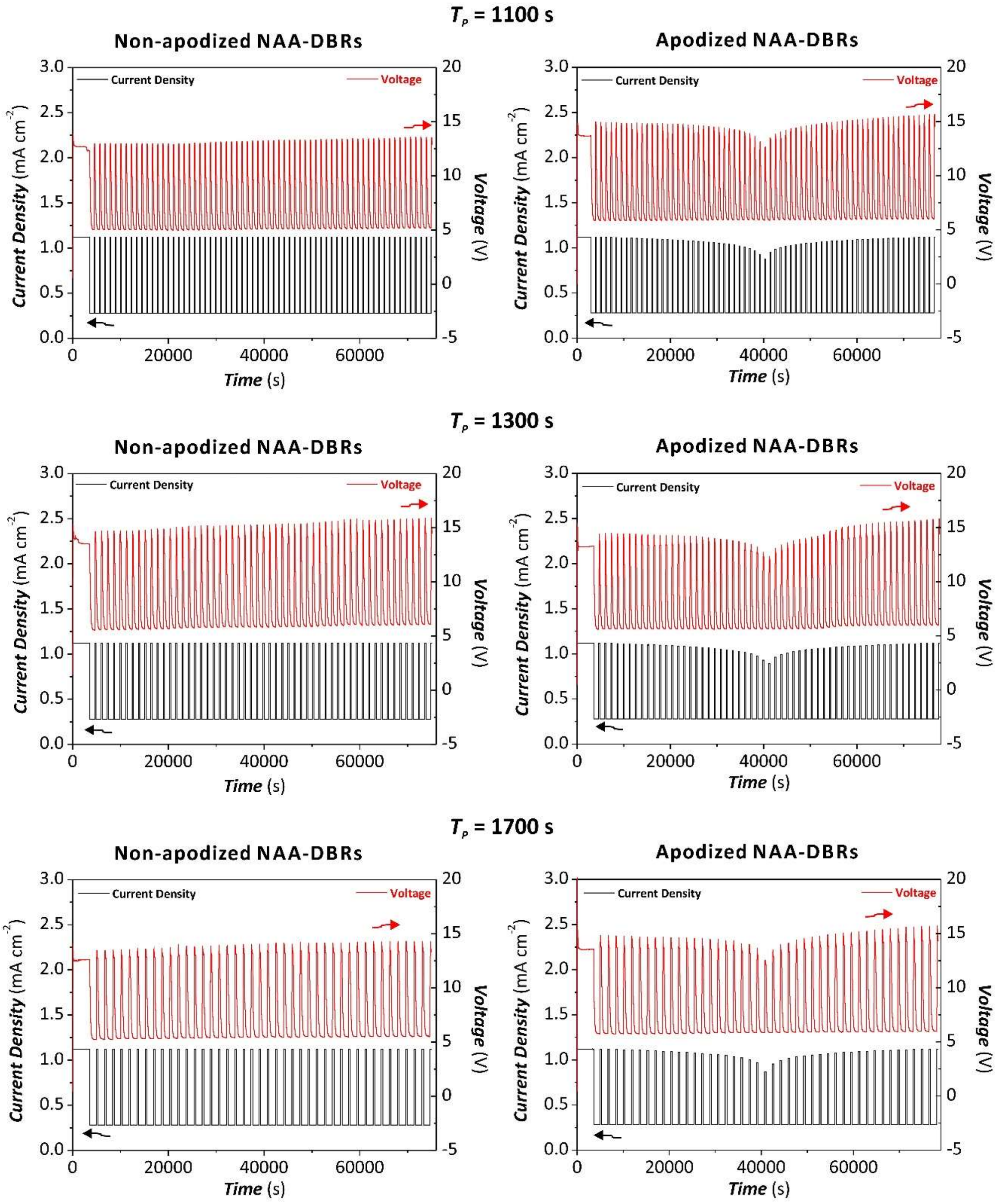

Figure S4. Anodization profiles of non-apodized and logarithmic negative apodized NAA-DBRs as a function of T_P.

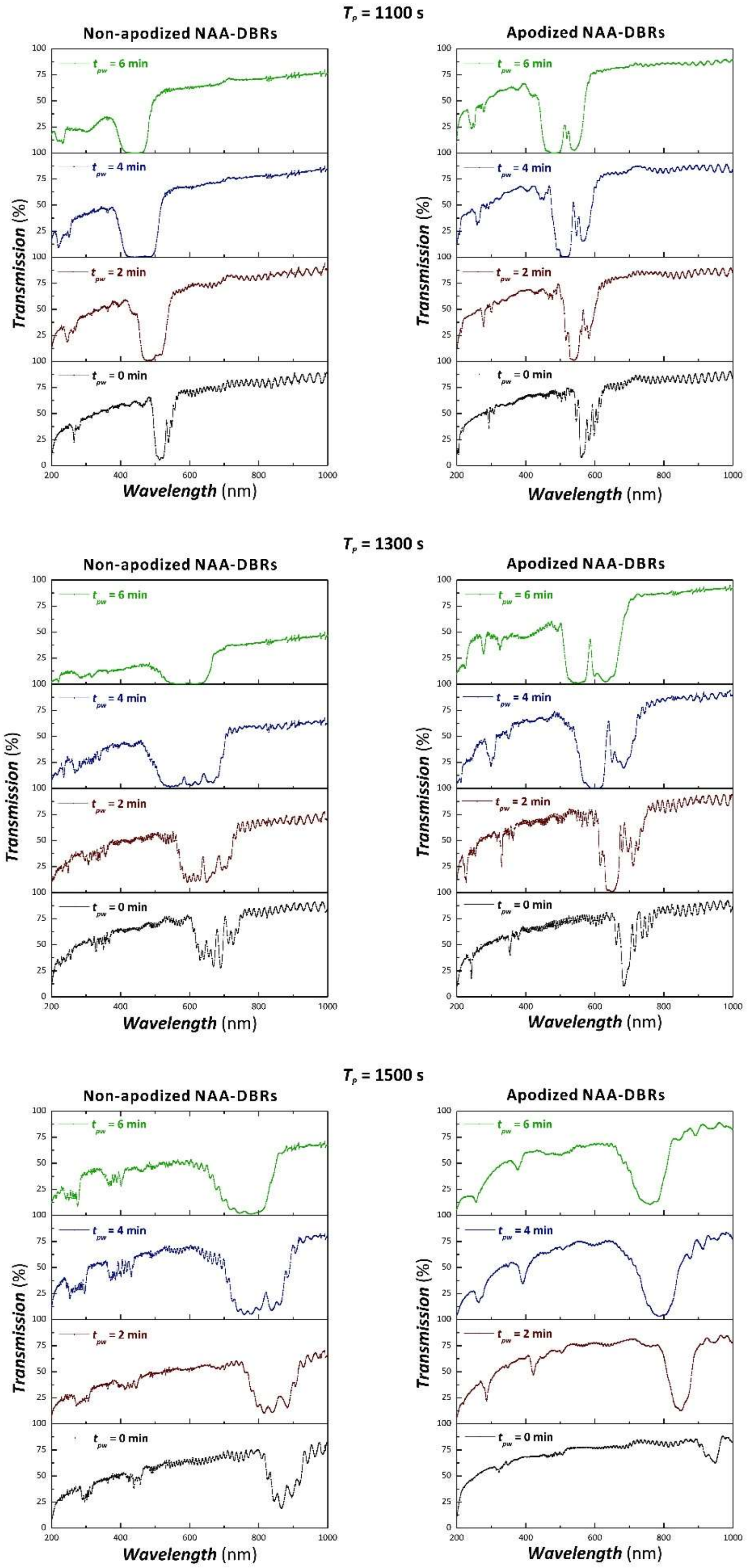

Figure S5. Transmission spectra of non-apodized and logarithmic negative apodized NAA-DBRs as a function of T_P and t_{pw}.

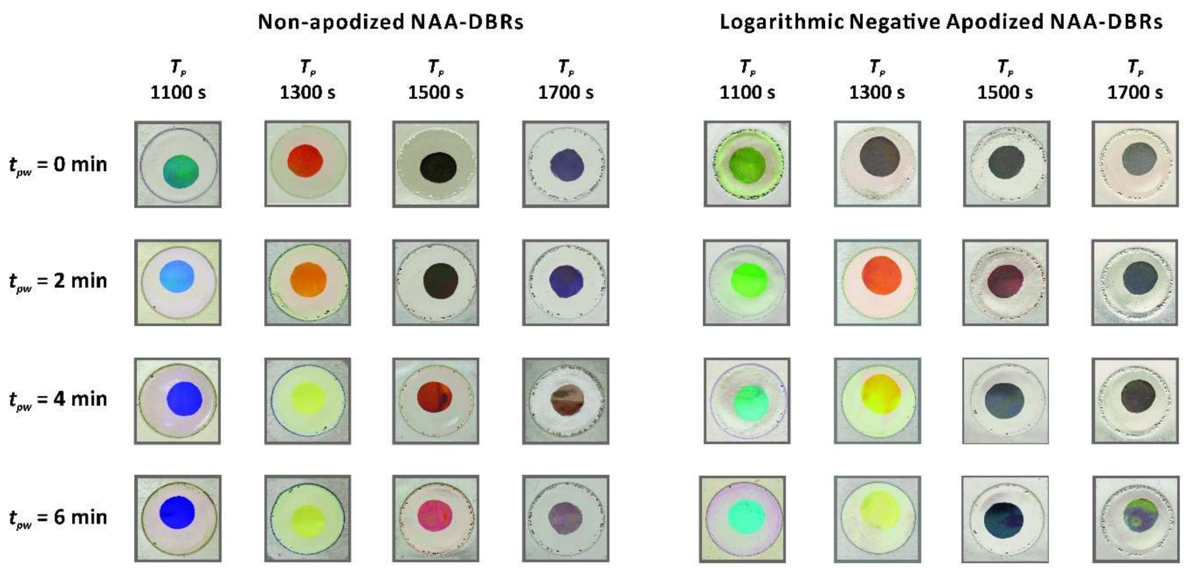

Figure S6. Digital pictures of non-apodized and logarithmic negative apodized NAA-DBRs as a function of T_P and t_{pw}.

The amplitudes as a function of time for the corresponding apodization functions applied in this study are given as **Equations S1 - S8:**

(i) Linear positive apodization

For $t \leq t_{an}/2$

$$A(t)_J = A_{min} + \left(\frac{A_{max} - A_{min}}{\frac{t_{an}}{2}}\right).t \qquad \textbf{Eq. S1}$$

For $t > t_{an}/2$

$$A(t)_J = A_{min} + \left(\frac{A_{max} - A_{min}}{t_{an} - \frac{t_{an}}{2}}\right).(t_{an} - t) \qquad \textbf{Eq. S2}$$

(ii) Linear negative apodization

For $t \leq t_{an}/2$

$$A(t)_J = A_{max} - \left(\frac{A_{max} - A_{min}}{\frac{t_{an}}{2}}\right).t \qquad \textbf{Eq. S3}$$

For $t > t_{an}/2$

$$A(t)_J = A_{max} + \left(\frac{A_{min} - A_{max}}{t_{an} - \frac{t_{an}}{2}}\right).(t - t_{an}) \qquad \textbf{Eq. S4}$$

(iii) Logarithmic positive apodization

For $t \leq t_{an}/2$

$$A(t)_J = A_{min} + \left(\frac{A_{max}-A_{min}}{\log\left(\frac{t_{an}}{2}+10\right)-1}\right).\left(\log(t+10)-1\right) \qquad \textbf{Eq. S5}$$

For $t > t_{an}/2$

$$A(t)_J = \left(\frac{A_{min}-A_{max}}{\log(t_{an}+10)-\mathrm{lo}\ \left(\frac{t_{an}}{2}+1\ \right)}\right).\left(\log(t+10)-\log\left(\frac{t_{an}}{2}+10\right)\right)+A_{max} \qquad \textbf{Eq. S6}$$

(iv) Logarithmic negative apodization

For $t \leq t_{an}/2$

$$A(t)_J = A_{max} + \left(\frac{A_{min}-A_{max}}{\log\left(\frac{t_{an}}{2}+10\right)-1}\right).\left(\log(t+10)-1\right) \qquad \textbf{Eq. S7}$$

For $t > t_{an}/2$

$$A(t)_J = \left(\frac{A_{max}-A_{min}}{\log(t_{an}+10)-\log\left(\frac{t_{an}}{2}+10\right)}\right).\left(\log(t+10)-\log\left(\frac{t_{an}}{2}+10\right)\right)+A_{min} \qquad \textbf{Eq. S8}$$
